# Paper-Based Biosensors for Monitoring Binding, Blocking, and Surrogate Neutralizing Antibody Responses Against Viral Infections

**DOI:** 10.3390/bios16080420

**Published:** 2026-08-04

**Authors:** Yiren Yin, Yujie Yi, Yazheng Yu, Tatyana Aleksandrovna Khrustaleva, Linlin Zhai, Jianhai Yu, Wei Zhao, Chenguang Shen

**Affiliations:** 1 BSL-3 Laboratory (Guangdong), Guangdong Provincial Key Laboratory of Tropical Disease Research School of Public Health, Department of Laboratory Medicine, Zhujiang Hospital, Southern Medical University, Ministry of Education, Guangzhou 510515, China; yinyiren1203@163.com (Y.Y.); yi.yujie@foxmail.com (Y.Y.); yyz102604@163.com (Y.Y.); zll15649856209@163.com (L.Z.); chienhai@163.com (J.Y.); zhaowei@smu.edu.cn (W.Z.); 2 Multidisciplinary Diagnostic Laboratory, Institute of Physiology, National Academy of Sciences of Belarus, 220072 Minsk, Belarus; tanissia.lir@gmail.com; 3 Key Laboratory of Infectious Diseases Research in South China, State Key Laboratory of Muti-Organ Injury Prevention and Treatment, Southern Medical University, Ministry of Education, Guangzhou 510515, China

**Keywords:** paper-based biosensors, antibodies, vaccine monitoring, lateral flow assay, microfluidic paper-based analytical devices, clinical validation

## Abstract

Virus-specific antibody responses, including binding antibodies and neutralizing antibodies (nAbs), are important indicators of antiviral immune status after infection or immunization. They provide complementary information on antiviral humoral immunity after infection or vaccination. Antigen-binding antibodies indicate previous exposure and the magnitude of the immune response, whereas receptor-blocking and functional neutralization assays assess whether antibodies interfere with viral entry or infection. Conventional neutralization assays, such as plaque reduction neutralization tests and pseudovirus neutralization tests, provide functional information but are labor-intensive, time-consuming, biosafety-restricted, and difficult to deploy for large-scale or decentralized monitoring. Paper-based biosensors, including lateral flow assays (LFAs), microfluidic paper-based analytical devices (μPADs), and paper-based ELISA, have emerged as promising point-of-care tools owing to their low cost, portability, simple operation, and compatibility with visual or digital readouts. This review critically evaluates these platforms according to whether they measure antigen-binding antibodies, receptor-blocking activity, surrogate neutralization, or functional neutralization and summarizes the applications of these three platforms for monitoring antibody responses against SARS-CoV-2, influenza, dengue, Zika, and monkeypox viruses. Unlike previous reviews that mainly focus on general paper-based biosensor design or conventional nAb assays, this review emphasizes the distinction between antigen-binding, receptor-blocking, and surrogate neutralization readouts, and critically discusses how paper-based signals should be interpreted in relation to functional immunity. We further analyze key translational challenges, including quantitative accuracy, antigen cross-reactivity, standardization, clinical validation, regulatory positioning, and real-world implementation. Future development should combine multiplex detection, standardized calibration, digital and AI-assisted interpretation, and clinically validated assay formats. Paper-based biosensors have considerable potential for decentralized antibody monitoring and public health surveillance, but their clinical utility depends on clear assay positioning and validation against appropriate functional or reference methods.

## 1. Introduction

### 1.1. Importance of Binding and Neutralizing Antibodies in Vaccine-Induced Immunity

Neutralizing antibodies (nAbs) are a functional subset of antibodies in antiviral humoral immune responses. Their major role is to directly inhibit viral infection. In general, nAbs act by binding to key viral surface proteins or functional epitopes, thereby blocking virus–receptor interactions, interfering with membrane fusion, preventing viral entry, or disrupting early steps of infection. Through these mechanisms, nAbs can reduce the ability of viral particles to enter host cells and spread further. Therefore, nAb levels are commonly regarded as an important indicator of protective immune status, particularly when the aim is to evaluate whether an antibody response has functional activity against viral infection [1,2].

Although the generation of neutralizing antibodies is generally regarded as a desirable outcome of antiviral humoral immunity, it should not be assumed that all viral infections induce timely, sufficient, or clinically protective nAb responses. In some infections, the route of viral spread, tissue tropism, incubation period, immune evasion strategies, or anatomical localization of viral replication may limit the opportunity for effective nAb generation before disease progression. For example, rabies virus can spread through peripheral nerves toward the central nervous system, where circulating antibodies have limited access once neurological disease develops. Similarly, in some localized cutaneous viral infections, local tissue infection and cell-to-cell spread may reduce the direct relevance of systemic nAb levels. Therefore, nAb monitoring should be interpreted in a virus-specific and pathogenesis-aware manner.

Binding antibodies refer to antibodies that recognize and bind to viral antigens. These antibodies may target viral surface proteins, structural proteins, or other antigenic epitopes, and their presence usually indicates prior viral exposure or the development of a virus-specific humoral immune response. Compared with functional neutralization assays, binding antibody assays are generally easier to perform, faster, and more suitable for large-scale serological surveys and population-level immune background assessment. However, antigen binding alone does not necessarily mean that an antibody can prevent viral infection. Some binding antibodies may recognize non-neutralizing epitopes or bind to viral antigens without affecting receptor engagement, membrane fusion, viral entry, or replication. Therefore, binding antibodies should not be automatically interpreted as protective antibodies [3].

Accordingly, binding antibody responses and neutralizing antibody activity should be clearly distinguished when interpreting antibody test results. Binding antibody detection is useful for determining whether a virus-specific immune response has been generated and for assessing the magnitude and breadth of humoral immunity. In contrast, nAb detection provides more direct information about whether antibodies can functionally block viral infection. Although binding antibody levels may correlate with neutralizing activity in some settings, this relationship is influenced by viral species, antigenic epitopes, assay formats, and prior exposure history, and it is not always stable or consistent. This distinction becomes particularly important in the context of viral antigenic variation, cross-reactivity, and co-circulation of related viruses, where binding antibody results alone may overestimate protective immunity. Therefore, differentiating binding antibody detection from functional nAb assessment, and validating antibody results with neutralization assays or surrogate neutralization formats when necessary, is essential for accurately interpreting antibody test results and evaluating antiviral immune status [4,5].

### 1.2. Current Strategies for Antibody and Neutralizing Antibody Assessment

Conventional methods for detecting and quantifying neutralizing antibodies mainly include the virus neutralization test (VNT) and the pseudovirus neutralization test (pVNT). Among these, the virus neutralization test and the pseudovirus neutralization test are regarded as classical methods or gold standards for evaluating the functional activity of nAbs. Such detection methods require incubation of serum samples from subjects with live virus or pseudovirus, followed by addition of the mixture to a cell culture system, and determining the inhibitory effect of antibodies in the sample on viral infection by observing changes in viral infectivity, cytopathic effects, or viral replication levels. These traditional methods provide relatively high specificity and sensitivity, more directly reflecting the neutralizing function of antibodies, and remain valuable in vaccine development, immunogenicity evaluation, and laboratory confirmation testing [1,2,6,7,8,9].

However, these methods also have clear limitations. First, live virus neutralization tests require laboratories with high biosafety levels, and detection of some viruses may even require BSL-3 conditions to ensure safe live virus operations. Second, these methods involve complex steps and relatively long turnaround times, requiring hours to days to generate results. Furthermore, the experimental process depends on cell culture, specialized equipment, and trained technical personnel, making broad implementation in primary healthcare institutions, remote areas, or resource-limited environments difficult. These limitations also constrain the deployment of traditional detection methods in large-scale population immune monitoring and rapid public health response [1].

To reduce biosafety risks associated with live-virus handling, the surrogate virus neutralization test (sVNT) has been developed. This method does not directly use live virus, but instead simulates the interaction between viral surface proteins and host cell receptors in vitro. Compared with traditional virus neutralization tests, sVNT offers improved safety and faster detection speed, and is easier to deploy across different settings [10].

The enzyme-linked immunosorbent assay (ELISA) can provide different types of antibody information depending on its assay format. Conventional antigen-coated ELISA primarily detects antigen-binding antibodies, including immunoglobulin G (IgG) and immunoglobulin M (IgM), and should not be equated with functional neutralization. By contrast, competitive or receptor-blocking ELISA formats can assess the inhibition of virus-receptor interactions and may therefore provide surrogate neutralization readouts. The distinctions among VNT or plaque reduction neutralization test, pVNT, sVNT or receptor-blocking ELISA, and conventional binding ELISA, in terms of what each assay measures, its functional relevance, and its main limitations, are summarized in Table 1.

Even so, the existing nAb testing system still cannot fully meet the need for rapid, broad-coverage, and low-cost immune monitoring. Although conventional laboratory assays are reliable, they remain limited by testing speed, operational convenience, cost, equipment dependence, and accessibility [11].

### 1.3. Growing Need for Rapid and Decentralized Immunity Monitoring

As global vaccination coverage expands and emerging and re-emerging viral diseases continue to threaten public health, the demand for immune monitoring is shifting from small-scale testing in traditional central laboratories toward rapid, decentralized, and large-scale population monitoring. Traditional nAb detection methods are increasingly unable to meet the current urgent need for POCT and decentralized testing [10,12].

Rapid and decentralized immune monitoring has substantial practical significance. First, substantial interindividual differences exist in immune responses after vaccination. Factors such as age, underlying diseases, immune status, previous infection history, vaccine type, and vaccination interval can all affect the strength and duration of nAb responses. Relying solely on a unified vaccination program makes it difficult to accurately judge the true immune protection status of different individuals. Moreover, nAb levels gradually decline over time, and dynamic monitoring can help identify populations with low antibody levels or faster immune waning, thereby informing booster vaccination strategies. Additionally, when viral variants emerge or outbreaks occur, rapidly understanding the immune status of different populations and regions helps guide vaccine resource allocation, protection of key populations, and public health interventions. An ideal nAb monitoring platform should be rapid, accurate, low-cost, easy to operate, require small sample volumes, produce easily readable results, and be suitable for on-site use. In resource-limited areas in particular, detection technology that can be performed outside central laboratory settings—in primary healthcare or field settings—would markedly improve the coverage of vaccine immune monitoring and the efficiency of public health response. Given this need, new nAb testing technologies suitable for point-of-care testing (POCT) have become a research focus [12,13,14,15,16].

### 1.4. Emergence of Paper-Based Biosensors for POCT

Paper-based biosensors have attracted considerable attention for their unique advantages and are considered a highly promising POCT platform. Paper-based biosensors use nitrocellulose membranes, filter paper, chromatography paper, or cellulose paper as substrates, and achieve rapid detection of target analytes by immobilizing antigens, antibodies, enzymes, nanomaterials, or other recognition elements. Paper-based materials are characterized by low material costs, simple fabrication processes, scalable production, strong portability, and the ability to operate without external power, making them especially suitable for testing applications in primary, field, and resource-limited environments [11,17,18,19,20].

It should be noted that portability and rapid operation are not intrinsic consequences of paper alone; similar miniaturized biosensing systems can also be constructed using glass, polymers, fabrics, or other porous substrates. The particular value of paper lies in the combination of capillary-driven flow, low-cost disposability, compatibility with biomolecule immobilization, ease of patterning, scalable manufacturing, lightweight storage, and suitability for instrument-free or minimally instrumented readout. Therefore, paper should be viewed not merely as a passive support, but as a functional substrate that integrates fluid transport, reagent storage, reaction localization, and signal readout in a low-cost format.

The porous structure and hydrophilicity of paper substrates enable capillary-driven fluid flow, thereby eliminating the need for external pumps, valves, or complex fluidic instrumentation, which simplifies the structure of detection devices. At the same time, paper-based platforms can support intuitive visual readout or instrument-free result interpretation, lowering the operational threshold. These characteristics give paper-based biosensors clear advantages in rapid, field-based detection of various biomarkers, and also make them suitable for monitoring vaccine-induced neutralizing antibodies [11,17,18].

For nAb detection, paper-based biosensors mainly include lateral flow assay (LFA), microfluidic paper-based analytical devices (μPADs), and paper-based ELISA. For example, LFAs built with paper-based microfluidic technology can achieve semi-quantitative detection of SARS-CoV-2 nAbs through competitive binding between the viral receptor binding domain (RBD) and the host receptor, providing rapid, visually interpretable colorimetric readouts without specialized equipment. In addition, combining fluorescent labels, quantum dot labels, smartphone readouts, and AI algorithms with paper-based platforms further improves detection sensitivity, quantitative capability, and user-friendliness [1,10,12,21].

These advances indicate that paper-based biosensors have the potential to transform the paradigm of nAb monitoring. They can provide rapid, accurate, low-cost, and easy-to-operate detection methods, thereby supporting individualized assessment of vaccine effectiveness and public health surveillance. More importantly, paper-based sensors can be combined with immunization programs for rapid, large-scale, and decentralized monitoring of vaccine-induced immunity levels, providing a basis for booster vaccination policies, vaccine resource allocation, and future pandemic preparedness [10,11,12,21,22].

Previous reviews have predominantly focused either on the engineering and analytical design of paper-based biosensors or on conventional laboratory methods for neutralizing antibody assessment. However, limited attention has been paid to the biological meaning of different paper-based antibody readouts. This review therefore classifies the reviewed platforms according to whether they measure antigen binding, receptor-blocking activity, surrogate neutralization, functional neutralization, or an indirect correlate of neutralization. This classification is essential because assays with similar paper-based formats may support fundamentally different claims regarding immune function, clinical interpretation, and regulatory positioning.

## 2. Fundamentals of Paper-Based Antibody and Surrogate Neutralization Assays

### 2.1. Structural Components of Paper-Based Biosensors

The structural basis of paper-based nAb detection platforms mainly includes paper-based materials, recognition elements, signal labels, fluid control structures, and readout systems. Different types of paper-based sensors differ in their specific structures, but their common characteristic is that they exploit the porous structure, protein immobilization capacity, and capillary flow characteristics of paper-based materials to achieve sample transport, immune reaction, and signal readout [11,17,18,20].

Paper-based materials are the core substrate of detection devices. Commonly used materials include nitrocellulose membranes, filter paper, chromatography paper, and cellulose paper. These materials possess unique physicochemical properties that make them suitable for biosensing applications. First, paper-based materials have a high specific surface area and abundant hydroxyl functional groups, which enable the effective immobilization of biomolecules such as antigens and antibodies. For nAb detection, this biochemical compatibility forms the basis for achieving specific recognition and sensitive detection. Additionally, the inherent hydrophilicity and capillary action of paper materials enable pump-free passive fluid transport, allowing samples and reagents to move rapidly and controllably within the device, thereby simplifying device structure and reducing energy consumption [17,18].

The performance of these paper substrates differs substantially across flow rate, pore size, protein immobilization capacity, background signal, mechanical strength, batch-to-batch consistency, and suitable platform, as summarized in Table 2. These differences should be considered when selecting a substrate for a specific paper-based antibody or surrogate neutralization assay.

The mechanical properties of paper also provide flexibility for sensor structure design. Paper is flexible, foldable, and biocompatible, and can be cut, folded, laminated, or patterned to construct two-dimensional or three-dimensional paper-based analytical devices. This property enables paper-based platforms to accommodate different geometric structures according to different detection requirements—for instance, single-channel test strips, multi-test-line structures, multi-reaction-zone chips, or three-dimensional origami microfluidic structures. Rapid prototyping and scalable manufacturing are especially valuable for large-scale production of disposable, low-cost detection devices [23,24,25,26].

Recognition elements determine the detection specificity of paper-based sensors. For nAb detection, commonly used recognition elements include viral surface proteins, receptor binding domains, host receptor proteins, anti-human IgG/IgM antibodies, and specific antigenic epitopes—for example, HA or NA antigens for influenza virus detection. Signal labels convert immune recognition events into visible or measurable readouts; common types include colloidal gold, fluorescent microspheres, quantum dots, SERS tags, enzymes, nanozymes, and electrochemically active materials. Results can be read by the naked eye, portable readers, smartphone image analysis, or electrochemical devices [1,21,27,28,29].

Paper-based materials also offer environmental sustainability. Since paper is derived from renewable materials and is biodegradable, it more closely approximates the principles of green chemistry than plastic or glass substrates; its lightweight, compact form, and ease of storage and transport also make it suitable for point-of-care testing in remote areas and resource-limited settings. Low-cost manufacturing processes, such as wax printing, screen printing, inkjet printing, and 3D-printed hydrophobic barriers, further improve the accessibility and large-scale application potential of paper-based biosensors [17,25,26].

Hydrophobic barriers define hydrophilic channels and reaction zones on paper and can be introduced by several methods, including wax printing, inkjet printing, screen printing, photolithography, laser cutting, plasma treatment, PDMS or silane treatment, 3D printing, and super-glue patterning. The choice of method determines the resolution, throughput, and compatibility with subsequent biomolecule immobilization.

The reliability of these barriers must be explicitly controlled. Key criteria include whether the barrier penetrates the full thickness of the paper, whether channel edges are sharp and well defined, whether the barrier resists leakage under pressure, whether channel width is reproducible across devices, whether flow rate is stable, and whether the pattern tolerates serum or whole-blood matrices and is stable under variations in humidity and temperature. Recommended quality-control steps include dye-flow leakage tests, flow-time measurements, microscopic inspection of barrier edges, batch-to-batch comparison, storage-stability testing, and validation with real samples.

### 2.2. Mechanisms of Antibody Detection and Neutralization Assessment

The core principle of paper-based antibody and neutralization-related assays is to convert specific antigen–antibody recognition, virus-receptor interactions, or antibody blocking effects into readable signals. Depending on the detection target and reaction mode, these modes can be summarized as binding antibody detection, competitive receptor-blocking assay, and surrogate neutralization detection [1,10]. A clear distinction should be made between binding antibody detection and neutralizing antibody detection. Binding antibodies refer to antibodies that recognize and bind to viral antigens, but this binding does not necessarily prevent viral entry or replication. In contrast, neutralizing antibodies are a functional subset of antibodies that inhibit infection by blocking virus–receptor interaction, membrane fusion, or other key steps in viral entry. Therefore, binding antibody levels may reflect exposure or vaccine-induced humoral responses, but they cannot be directly interpreted as protective neutralizing activity unless validated against functional neutralization assays [3].

Binding antibody detection is the most basic mode of paper-based immunoassay. The fundamental principle is to immobilize viral antigens on the paper substrate or detection line, capture specific antibodies against that antigen in the sample, and then generate color, fluorescence, chemiluminescence, or electrical signals through labeled antibodies or labeled antigens. This method is simple and rapid, and is suitable for determining whether specific humoral immune responses have been generated after vaccination or natural infection. Paper-based ELISA and some LFAs often adopt this mode. Its limitation is that binding antibodies do not necessarily have neutralizing activity, so binding antibody detection can only indirectly reflect the magnitude of the immune response and cannot fully represent the level of protective neutralizing antibodies [17,30,31].

Competitive receptor-blocking assay more closely reflects the functional evaluation of nAbs. The protective effect of nAbs depends on their ability to block the binding of viral surface proteins to host cell receptors, thereby inhibiting viral entry into cells. Paper-based detection can exploit this mechanism, reconstituting the binding process between viral antigens and host receptors on a paper-based platform. When nAbs are present in the sample, these antibodies bind to key regions of the viral antigen and inhibit the binding of the viral antigen to the receptor or labeled probe, thereby causing changes in the detection signal [1,10].

Surrogate neutralization detection simulates key molecular interactions in the viral infection process without using live virus or cell culture. This mode is essentially the miniaturization and translation of the traditional surrogate virus neutralization test onto a portable paper-based platform. Compared with live virus neutralization tests, surrogate neutralization detection does not involve virus culture, has lower biosafety risks, is faster, and is more suitable for on-site applications. Its main advantage is that it can reflect, to some degree, the ability of antibodies to block virus-receptor binding, and is therefore closer to functional neutralization assessment than simple binding antibody detection. It remains, however, a simplified simulation of the actual viral infection process, and therefore needs to be validated for correlation with virus neutralization tests, pseudovirus neutralization tests, or clinical protection data [1,10].

Paper-based antibody assay detection does not detect only a single antibody form; depending on the platform design, it can detect binding antibody signals, receptor-blocking activity, or surrogate neutralization activity. Improving the correlation between paper-based detection results and true functional nAb levels is a key direction for the continued development of this field [1,10,16].

### 2.3. Signal Generation and Detection Modalities

The signal generation method of paper-based biosensors directly determines their sensitivity, quantitative capability, instrument dependence, and application scenarios. Signal modes used for nAb detection mainly include colorimetric detection, fluorescence detection, chemiluminescence detection, electrochemical detection, and photoelectrochemical detection [20,31,32].

Colorimetric detection is the most intuitive and widely used detection method in paper-based sensors, and is especially common in LFAs. This method relies on visible color changes caused by the presence of the target analyte; results can be observed by the naked eye or smartphone camera without complex instruments, making it particularly suitable for POCT and resource-limited environments. Commonly used colorimetric labels include gold nanoparticles, silver nanoparticles, carbon nanoparticles, and polystyrene microspheres. Among these, gold nanoparticles, due to their surface plasmon resonance properties, can produce distinct red or blue color signals and are the most commonly used labels in traditional LFAs. In SARS-CoV-2 nAb detection, gold nanoparticles can be conjugated to the viral RBD protein, and semi-quantitative assessment of nAb levels can be made through changes in signal intensity [1,33].

To improve the sensitivity of colorimetric detection, researchers have developed several signal amplification strategies. The silver enhancement method deposits silver ions on the surface of gold nanoparticles, thereby increasing particle size and enhancing the color signal; enzyme-catalyzed strategies use horseradish peroxidase and other enzymes to catalyze chromogenic substrates such as TMB, producing stronger color reactions; nanozymes, due to their enzyme-like catalytic activity along with better stability and lower cost, have also been used to enhance paper-based colorimetric detection signals. In addition, gradient detection designs and conjugate pad structures can also increase detection capability for low-titer antibodies by extending the contact time between the analyte and the labeled probe and increasing reaction opportunities. The main disadvantage of colorimetric methods is limited sensitivity and dynamic range; low-concentration samples may produce false negatives, and visual interpretation is easily affected by subjective factors and ambient light. The introduction of smartphone image analysis can improve result objectivity through color-intensity quantification, moving traditional colorimetric LFAs from qualitative detection to semi-quantitative or quantitative analysis [29,31,34].

Fluorescence detection uses quantum dots, fluorescent dyes, fluorescent microspheres, or upconversion nanoparticles as signal labels. Compared with colorimetric methods, fluorescence detection offers higher sensitivity, a wider dynamic range, and stronger quantitative capability. Quantum dots have high photostability and tunable emission spectra, suitable for multiplex detection; upconversion nanoparticles can absorb near-infrared light and emit visible fluorescence, reducing background interference from biological samples and improving the signal-to-noise ratio. The disadvantage of fluorescence detection is that it usually requires an excitation light source and a portable reader, so equipment requirements are higher than for ordinary colorimetric detection [21,35,36].

Chemiluminescence detection is based on enzyme-catalyzed reactions that generate light signals, requiring no external excitation light source. Commonly used enzymes such as horseradish peroxidase can catalyze substrates such as luminol to produce chemiluminescence signals. This method has low background noise and high sensitivity, and can reduce problems of autofluorescence and photobleaching encountered in fluorescence detection, making it suitable for quantitative detection of low-concentration antibodies. Combining portable chemiluminescence reading devices with paper-based platforms can help improve detection sensitivity and quantitative performance while retaining the advantages of rapid, low-cost, and simple LFAs [16,37].

Electrochemical detection converts antigen–antibody binding events into measurable electrical signals by directly integrating electrodes into paper-based materials. Commonly used electrodes include carbon electrodes and gold electrodes, which can be prepared by screen printing, inkjet printing, or sputtering. When neutralizing antibodies bind to viral antigens immobilized on the electrode surface, the electron transfer kinetics at the electrode interface are altered, causing changes in impedance, current, or potential. This method has the advantages of sensitivity, portability, ease of miniaturization, suitability for multiplex detection, and compatibility with digital readouts. Since electrical signals are naturally convenient for data collection and transmission, paper-based electrochemical sensors are particularly suitable for combining with smartphones, wireless communication modules, and cloud systems for remote immune monitoring and epidemiological surveillance [20,38,39].

Photoelectrochemical detection combines photochemical and electrochemical principles, using semiconductor materials such as titanium dioxide and cadmium sulfide as photoactive components. Under light excitation, semiconductors generate electron-hole pairs and form photocurrent; the presence of target antibodies or enzyme reaction products affects the charge transfer process at the electrode interface, thereby changing the photocurrent intensity. This method has high sensitivity, low background, and strong resistance to interference. Combining photoelectrochemical detection with μPADs can achieve rapid, sensitive detection of biomarkers related to infectious diseases, and provides a new technical direction for future nAb monitoring [40,41].

Overall, different signal modes have their respective strengths. Colorimetric detection is most suitable for low-cost, rapid screening; fluorescence and chemiluminescence detection help improve sensitivity and quantitative capability; electrochemical and photoelectrochemical detection are better suited for intelligent, digital, and remote monitoring. With the development of nanomaterials, signal amplification technologies, portable reading devices, and AI algorithms, paper-based nAb detection is transitioning from simple qualitative judgment to sensitive, quantitative, connectable, and scalable monitoring [20,21,22,33,39,42].

## 3. Lateral Flow Assays for Antibody and Surrogate Neutralization Monitoring

### 3.1. Principle of LFA

Lateral flow assay (LFA) is one of the most widely used forms of paper-based biosensors, with main advantages of simple operation, rapid detection, and relatively low cost. The basic working principle of LFA is to combine antigen–antibody specific immune recognition with capillary flow on a paper-based membrane, enabling the sample to move directionally along the test strip without external pumps or complex instruments, and generating a signal visible to the naked eye or readable by instruments in the detection zone [28,43].

**Figure 1:**
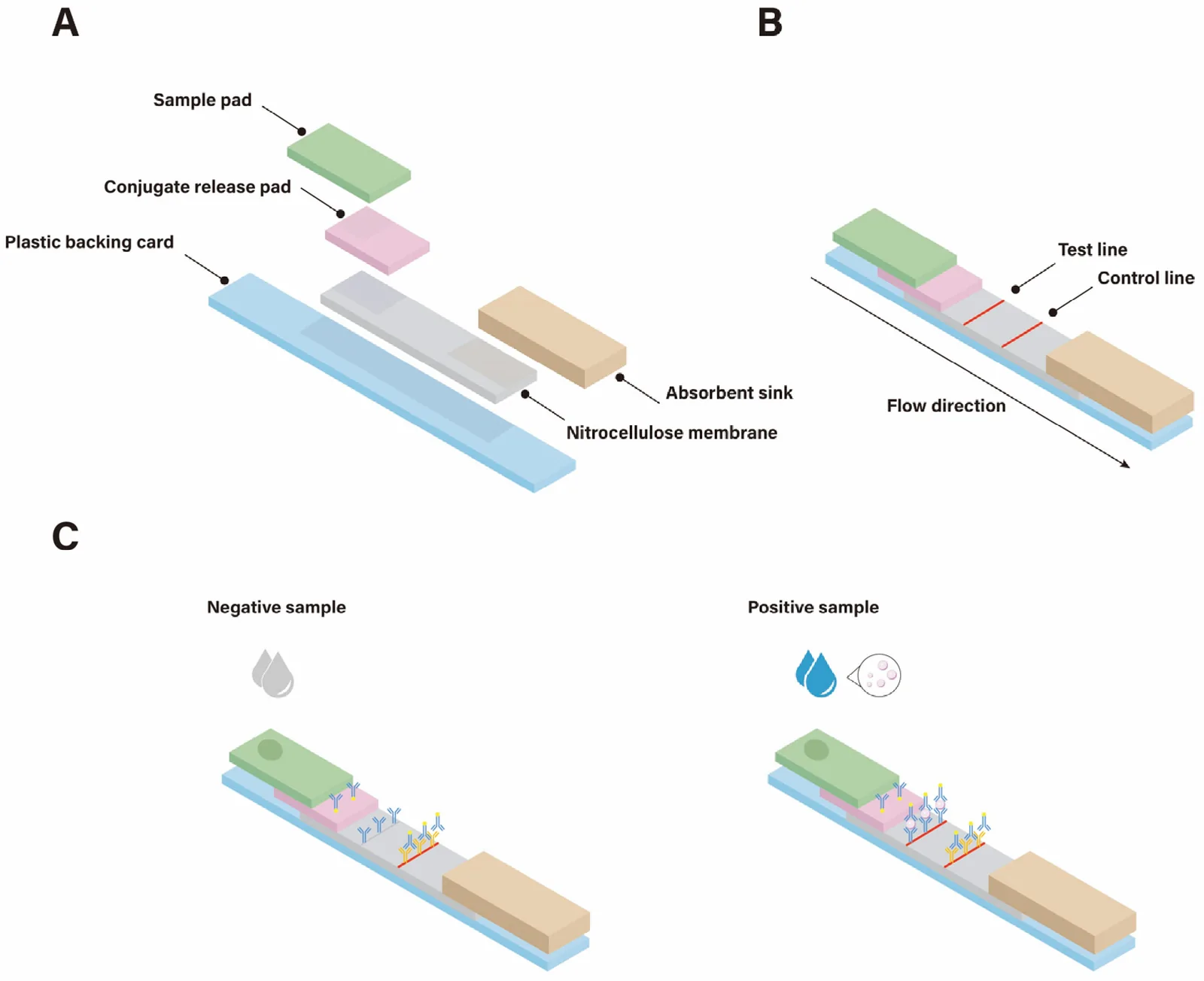
Schematic illustration of an antibody-detection lateral flow assay based on a sandwich-type format. (**A**) Exploded view of the LFA strip components. (**B**) Assembled LFA strip. (**C**) Representative antibody-positive and antibody-negative readouts in a sandwich-type antibody detection system. This figure illustrates antibody detection specifically and does not represent all possible LFA formats, such as competitive LFAs used for small-molecule detection [55].

Traditional LFA test strips are composed of five functional modules assembled on a plastic backing card: a sample pad, conjugate (release) pad, nitrocellulose membrane (NC membrane), absorbent sink, and backing card (Figure 1A). After the sample is applied to the sample pad, the liquid moves downstream through the conjugate pad driven by capillary action. In the conjugate pad, the sample rehydrates pre-dried labeled detection antibodies or labeled antigens; these labels can be colloidal gold nanoparticles, fluorescent microspheres, quantum dots, or other signal reporters. If the target analyte is present in the sample, it binds to the labeled probe to form an immune complex, which then migrates further along the NC membrane (Figure 1B) [28,43].

When the complex reaches the test line (T line) region on the NC membrane, the capture molecules immobilized at this site recognize and bind to the immune complex, causing localized accumulation of the signal reporter and generating a visible signal. In traditional sandwich-type detection, the target analyte is jointly recognized by the labeled detection antibody and the capture antibody, forming a “sandwich” structure that traps the reporter at the T line and produces a colorimetric or fluorescent signal—corresponding to a positive result (Figure 1C, right). Downstream of the T line, the control line (C line) captures any excess unbound labeled probe via secondary antibodies, producing an additional colored band that serves as an internal validity control. Regardless of target analyte presence, the C line must develop color to confirm proper sample migration, reagent release, and reaction integrity; in the absence of analyte, only the C line appears—indicating a negative result (Figure 1C, left). This dual-line design makes the C line an essential structural element for judging assay validity [28,43].

For antibody and neutralization-related assessment, LFAs can adopt different detection modes. The binding antibody detection mode is mainly used to determine whether specific antibodies against viral antigens are present in the sample; the competitive blocking mode aligns more closely with functional evaluation of neutralizing antibodies. Taking SARS-CoV-2 as an example, the detection system can be constructed using the interaction between RBD and ACE2. When neutralizing antibodies capable of binding RBD are present in the sample, they can block the binding of RBD to ACE2, thereby changing the signal intensity at the detection line. This mode does not require live virus or cell culture, and can simulate the key molecular events of virus entry into host cells on a paper-based platform, making it suitable for rapid neutralizing antibody screening [1,10,12,44]. Accordingly, LFA results should be interpreted according to assay format. Antigen-binding LFAs mainly indicate the presence of virus-specific binding antibodies, whereas competitive RBD-ACE2 or similar blocking LFAs provide a surrogate readout that is more closely related to neutralizing activity.

The choice of labeling agent has an important influence on the sensitivity and specificity of LFAs. Traditional colloidal gold nanoparticles provide a visible red signal, enabling rapid and intuitive interpretation. With the development of nanotechnology, new labels such as fluorescent microspheres, quantum dots, upconversion nanoparticles, and surface-enhanced Raman scattering (SERS) tags have been introduced into LFA, markedly improving detection sensitivity and supporting quantitative analysis. The detection limit of SERS-labeled LFA can be substantially lower than that of traditional colloidal gold methods, aiding the detection of low-concentration antibodies and improving early serological diagnostic capability [27,45].

The nitrocellulose membrane is the core component of LFA, and its pore size, protein adsorption capacity, hydrophilicity, and flow rate directly affect detection kinetics, signal intensity, and sensitivity. Membrane material optimization—for example, nitrocellulose/cotton fiber composite materials—can improve flow rate and protein binding efficiency; treatment to render the detection and control line regions transparent can also improve chemiluminescence LFA signal detection by reducing light scattering. Buffer salts and surfactants in the sample pad help improve sample flow and reduce non-specific binding; the conjugate pad stores and releases dry labeled probes; the absorbent pad maintains capillary flow by continuously absorbing liquid, enabling the reaction to proceed smoothly [37,46].

LFA design also needs to address some typical problems, such as the “hook effect.” When the concentration of the target analyte in the sample is too high, excess analyte may saturate the binding sites and impede the formation of the sandwich structure, leading to false negative results. To mitigate this problem, the release time of labeled probes can be controlled through an intermediate pad, or delayed release can be achieved using soluble materials such as polyvinyl alcohol, thereby expanding the detection dynamic range and improving accuracy in high-concentration samples [47].

Overall, LFA combines antigen–antibody specific recognition, paper-based capillary flow, and signal labeling technology through a modular test strip structure to achieve rapid, low-cost, user-friendly detection of target analytes. With continuous advances in labeling technology, membrane materials, flow control strategies, and readout methods, the sensitivity, specificity, and quantitative capability of LFA continue to improve, and its scope of application has expanded from infectious disease diagnosis to food safety, environmental monitoring, and vaccine-induced antibody monitoring [28,43,45,48,49,50,51,52,53,54].

### 3.2. Applications of LFA

The application of LFA in vaccine-induced neutralizing antibody detection is most clearly represented by SARS-CoV-2 research. During the COVID-19 pandemic, rapid and accessible antibody detection tools were needed for assessing immune status after vaccination, monitoring antibodies after infection, judging the risk of breakthrough infections, and formulating booster vaccination strategies. LFAs based on the interaction between SARS-CoV-2 RBD and ACE2 can detect antibodies with potential neutralizing activity through competitive binding or blocking mechanisms. If neutralizing antibodies are present in the sample, they can block RBD-ACE2 binding, thereby changing the signal at the detection line, enabling rapid semi-quantitative or quantitative assessment [10,12,15,16,44,56,57].

In SARS-CoV-2 neutralizing antibody detection, LFAs can use different signal systems such as colloidal gold, fluorescent microspheres, quantum dots, or SERS labels. Colloidal gold LFAs are suitable for rapid screening, with results directly readable by the naked eye; fluorescence and quantum dot LFAs can improve sensitivity and dynamic range; the introduction of smartphone readouts and AI algorithms can further improve result objectivity and quantitative capability. These technologies enable LFAs to be applied for dynamic monitoring of antibody levels after vaccination, identification of populations with low antibody levels, screening of convalescent plasma, and public health population immune surveys [21,22,27,29,33].

The application of LFA extends beyond SARS-CoV-2. For influenza virus, vaccine protection efficacy is closely related to antibodies against hemagglutinin (HA) and neuraminidase (NA). Since influenza virus antigens vary frequently and vaccine strains need to be updated regularly, rapid monitoring of antibody responses to different subtypes is important for evaluating vaccine efficacy and guiding vaccine strain selection. LFAs can detect specific antibodies generated after vaccination by immobilizing HA, NA, or related recombinant antigens, providing a rapid field tool for influenza vaccine immune monitoring [28,43].

For flaviviruses such as dengue virus and Zika virus, LFAs can be used for antibody screening and serological surveys, but their application faces more complex cross-reactivity issues. There is strong antigenic similarity between different dengue virus serotypes and between dengue virus and Zika virus, which may lead to false positive results or difficulty in distinguishing the source of infection. The design of flavivirus-related LFAs therefore needs to select more specific antigenic epitopes, and may be combined with competitive detection or multiplex detection modes to improve the ability to distinguish antibodies against different viruses or serotypes [23].

For monkeypox virus, the recent increase in global transmission has heightened the demand for monitoring vaccine-induced antibodies. Antibody levels, duration of protection, and need for booster immunization in populations vaccinated with vaccines such as MVA-BN still require further evaluation. LFAs based on monkeypox virus-specific antigens, such as the A29L protein, have the potential for rapid detection of specific antibody responses after vaccination or natural infection, and can serve population immune level assessment, vaccine distribution, and booster vaccination strategy formulation during outbreaks [58,59,60].

Beyond viral vaccine monitoring, LFAs are also widely used in infectious disease diagnosis, food safety, environmental monitoring, and animal disease detection. For example, colloidal gold-labeled protein A/G can be used for Brucella antibody detection, competitive LFAs can be used for detection of canine parvovirus antibodies, and SERS- or fluorescent-labeled LFAs can be used for detection of low-concentration antibodies or antigens. These applications show that LFA has good platform adaptability and scalability. By replacing antigens, antibodies, and signal labels, the same basic platform can be adapted for use with different pathogens and different detection targets [27,45,61].

In terms of practical application scenarios, LFAs are particularly suitable for primary healthcare institutions, community screening, vaccination sites, schools, border checkpoints, and resource-limited areas. Their detection process is simple, results are rapid, and equipment dependence is low, supporting large-scale population screening and field decision-making. With the development of portable readers, smartphone analysis, and AI algorithms, the application of LFA in vaccine-induced neutralizing antibody monitoring is expanding from simple qualitative screening to semi-quantitative, quantitative, and digital immune monitoring [28,33,43].

### 3.3. Advantages and Limitations of LFA

The main advantages of LFA lie in its speed, low cost, portability, and ease of operation. Compared with traditional virus neutralization tests, LFAs do not require live virus, cell culture, or high-level biosafety laboratories, and most detections can be completed within 15–30 min, substantially reducing detection time. The operational procedure is simple, with relatively low requirements for user expertise, and can be completed by primary healthcare workers or even non-professionals with simple training, making it particularly suitable for POCT, field screening, and resource-limited environments [10,12,28,43,44].

LFA has a simple structure and is easy to scale up for production. Test strips are composed of multiple functional modules, with mature manufacturing processes, enabling standardization and single-use disposability. Paper-based materials themselves are inexpensive, lightweight, and easy to store and transport, suitable for large-scale deployment. The ability to obtain results without complex instruments enables application in primary healthcare institutions, communities, schools, border checkpoints, and epidemic sites, helping to expand the coverage of neutralizing antibody monitoring [11,28,43].

LFA also exhibits strong platform plasticity. By replacing capture antigens, detection antibodies, or labels, the same platform can be applied to the detection of different viruses and different antibody types. For pathogens such as SARS-CoV-2, influenza virus, dengue virus, Zika virus, and monkeypox virus, LFAs can be designed based on viral surface antigens or receptor binding characteristics. Its detection mode is also relatively flexible—capable of adopting binding antibody detection, double antigen sandwich detection, or competitive blocking detection—adapting to different neutralizing antibody monitoring needs [23,28,43,59].

With advances in labeling materials and readout technologies, LFA performance continues to improve. Traditional colloidal gold LFAs can achieve low-cost naked-eye interpretation; fluorescent microspheres, quantum dots, and SERS labels can markedly enhance detection sensitivity and quantitative capability; nanozymes, silver enhancement, and photothermal signal strategies can further amplify signals; smartphone and AI image analysis can reduce subjective interpretation errors and improve result consistency and digitization. These advances are driving LFAs from traditional qualitative detection toward semi-quantitative, quantitative, and intelligent detection [21,22,27,29,31,33].

LFA also has certain limitations, however. First, traditional LFAs are mostly still qualitative or semi-quantitative, making it difficult to provide continuous quantitative functional indicators such as IC50 and IC90 like virus neutralization tests. For scenarios requiring precise assessment of individual immune protection levels or comparison of immune efficacy between different vaccines, LFA results alone may be insufficient. Second, many LFAs detect binding antibodies or surrogate blocking capability rather than functional neutralization in the actual virus infection process. LFA results therefore need to be validated for correlation with virus neutralization tests, pseudovirus neutralization tests, or surrogate neutralization tests before being used more reliably for vaccine protection efficacy evaluation [1,2,16,57].

Second, LFA sensitivity may still be lower than that of traditional ELISA or neutralization tests in some cases. For early post-vaccination samples, samples taken long after vaccination, immunocompromised populations, or samples with low antibody titers, traditional colorimetric LFAs may produce false negatives. Although fluorescence, SERS, and nanomaterials can improve sensitivity, they may also increase cost, equipment dependence, and operational complexity. Improving detection performance requires balancing sensitivity, cost, portability, and ease of use [21,27,29,62].

In addition, the stability of LFA results may be affected by multiple factors. Nitrocellulose membrane pore size, protein adsorption capacity, batch-to-batch differences in paper-based materials, sample matrix, temperature, humidity, storage conditions, and readout methods can all affect flow rate, reaction efficiency, and signal intensity. Cellular components, hemoglobin, or other interfering substances in whole blood samples may also affect fluid migration and result interpretation. For colorimetric LFAs read by naked eye, subjective judgment by the operator and ambient light can also introduce errors [19,37,46,63].

Finally, for multiplex detection and detection of viruses with similar antigens, LFAs still face spatial limitations, cross-reactivity, and signal interference issues. The number of detection lines is limited, and if multiple antibodies or multiple viral subtypes are detected simultaneously, the test strip structure and result interpretation become complex. For pathogens with similar antigens such as dengue virus and Zika virus, cross-reactivity may lead to false positives or difficulties in result interpretation. Future efforts are therefore needed to improve the reliability of LFA in neutralizing antibody monitoring through optimized antigen epitope selection, improved membrane materials, strengthened flow control design, established standardized calibration systems, and conducted clinical validation [23].

LFA is a paper-based detection platform suitable for rapid screening and field immune monitoring, with clear advantages in speed, cost, and accessibility, but cannot yet fully replace traditional neutralization tests. With the development of high-performance signal labels, portable reading devices, AI analysis, and standardized validation systems, LFA is expected to contribute more substantially to vaccine-induced neutralizing antibody monitoring, large-scale population immune assessment, and public health emergency response [28,33,43].

## 4. Microfluidic Paper-Based Analytical Devices (μPADs)

### 4.1. Principle and Design Strategies of μPADs

Microfluidic paper-based analytical devices (μPADs) are a class of miniature analytical platforms that use paper-based materials as substrates and rely on capillary action to drive fluid flow. Unlike traditional laboratory detection, which depends on pumps, tubing, and large instruments, μPADs achieve passive transport of samples and reagents through the porous structure of paper itself. Consequently, they offer low cost, portability, ease of operation, and suitability for field testing. Noviana et al. systematically summarized the development of μPADs from device design to practical application, noting that paper-based microfluidic platforms can control the direction of liquid flow through hydrophilic channels and hydrophobic barriers, thereby completing steps such as sample transport, mixing, reaction, and detection on paper [20]. Hou et al. also highlighted the considerable value of paper-based detection devices in point-of-care testing (POCT) scenarios owing to their inexpensive materials, simple manufacturing, and rapid detection [11].

**Figure 2:**
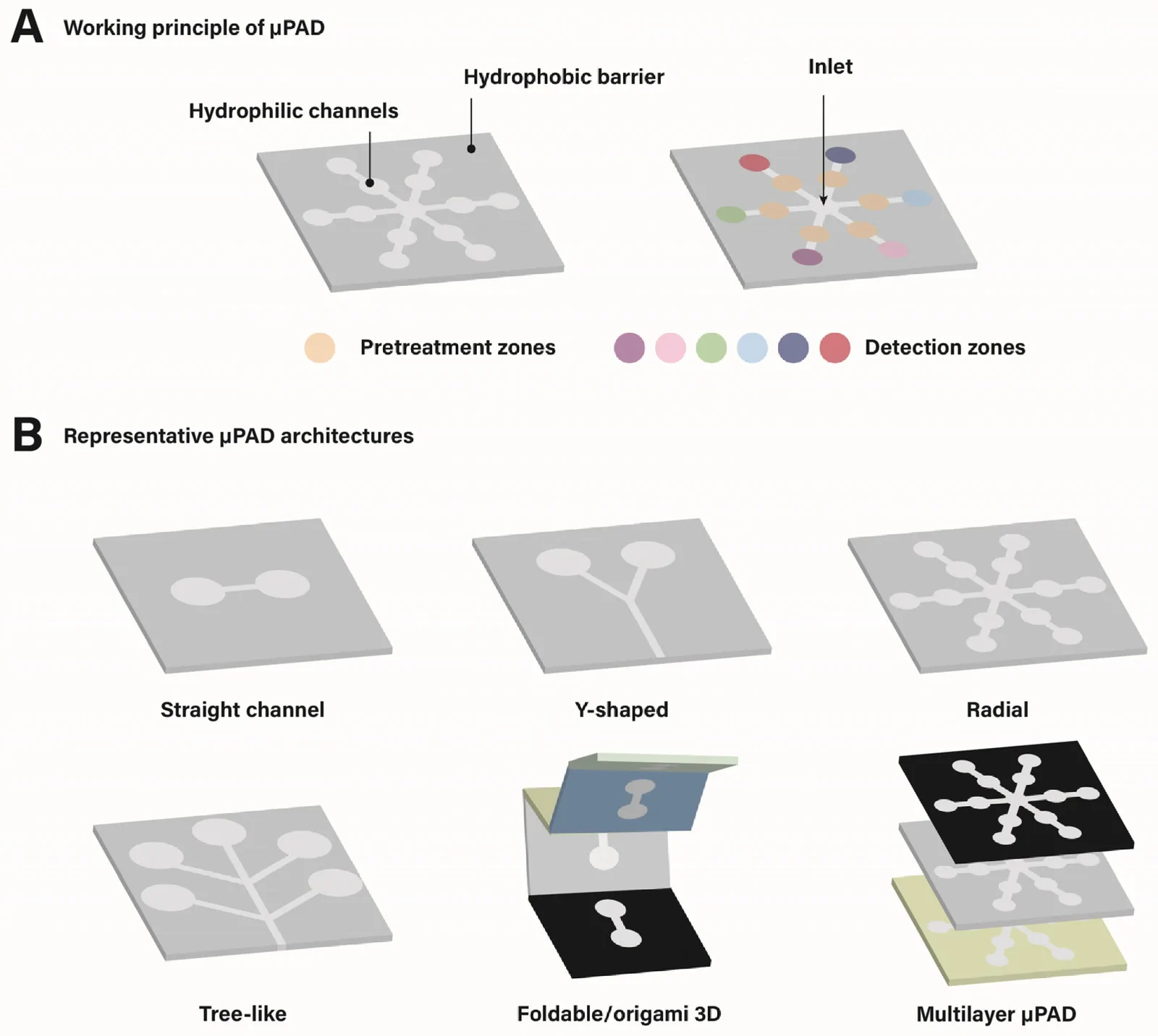
Working principle and representative architectures of microfluidic paper-based analytical devices (μPADs). (**A**) Working principle of a μPAD. Hydrophilic channels are defined on a paper substrate and surrounded by hydrophobic barriers, which guide the sample from the inlet through pretreatment zones to multiple detection zones by passive capillary flow. (**B**) Representative μPAD architectures. From left to right and top to bottom: straight-channel, Y-shaped, radial, and tree-like two-dimensional layouts; foldable/origami three-dimensional design; and multilayer μPAD configuration.

The basic structure of a μPAD typically comprises a sample introduction zone, microfluidic channels, reaction zones, detection zones, and absorbent zones. The core design concept is to construct controllable liquid-flow paths on paper-based materials so that samples reach different reaction zones along preset routes. Figure 2A schematically illustrates this working principle: hydrophilic channels are patterned on a paper substrate and surrounded by hydrophobic barriers that confine sample flow to predefined paths by capillary action. Once the sample is introduced through the inlet, it sequentially passes through pretreatment zones and reaches multiple detection zones for parallel readout. Figure 2B summarizes representative μPAD architectures, including straight-channel, Y-shaped, radial, and tree-like two-dimensional designs, as well as foldable/origami three-dimensional and multilayer configurations that provide enhanced liquid handling and stability.

A variety of patterning methods can be used to fabricate these devices, including wax printing, inkjet printing, photolithography, laser cutting, and 3D printing. Kang et al. proposed a low-cost method for fabricating paper-based analytical devices using super glue, indicating that μPAD processing is moving toward simpler, cheaper, and more easily deployable fabrication routes [26]. Catalan-Carrio et al. explored the structural design of μPADs from the perspective of channel-size prediction, suggesting that channel width, length, and material properties directly affect liquid-flow speed and detection stability [64]. In addition, the three-dimensional μPADs constructed by Jeon et al. reduce evaporation through closed channels, improving the stability of paper-based detection and demonstrating that three-dimensional structural design helps overcome the limitations of traditional two-dimensional paper-based devices in liquid control [24].

For neutralizing antibody detection, the value of μPADs lies mainly in their ability to simplify and integrate complex immune reaction processes onto a paper-based platform. The key to neutralizing antibody detection is not simply to determine whether antibodies are present, but to determine whether antibodies have the function of blocking viral infection. Taking SARS-CoV-2 as an example, the binding of the viral Spike protein RBD to the host ACE2 receptor is an important step in viral cell entry, and neutralizing antibodies can block this binding process. μPADs based on the RBD–ACE2 blocking principle can therefore indirectly evaluate the neutralizing antibody level in a sample without using live virus or cell culture. The semi-quantitative paper-based microfluidic surrogate virus neutralization test developed by Mahmud et al. is a typical study combining SARS-CoV-2 neutralizing antibody detection with the μPADs platform [1].

In addition, μPADs can be combined with various signal readout modes, including colorimetric, fluorescence, chemiluminescence, electrochemical, photothermal, and nanomaterial-enhanced signals. Selvakumar and Kathiravan reviewed the sensing materials in μPADs, pointing out that different sensing materials affect detection sensitivity, signal stability, and application scenarios [32]. Pang et al. further summarized the role of nanomaterials in improving the detection performance of paper-based analytical devices, indicating that nanomaterials can improve the sensitivity and quantitative capability of paper-based detection platforms by enhancing optical, electrochemical, or catalytic signals [32]. μPADs are therefore not simply paper fluidic channels, but comprehensive detection platforms that can be combined with multiple signal amplification strategies.

### 4.2. Applications of μPADs in Neutralizing Antibody and Viral Immune Monitoring

Currently, μPAD research for vaccine-induced neutralizing antibody detection is most representative for SARS-CoV-2. The semi-quantitative paper-based microfluidic surrogate virus neutralization test reported by Mahmud et al. introduced the RBD–ACE2 blocking reaction into a paper-based microfluidic platform for detection of SARS-CoV-2 neutralizing antibodies [1]. This method does not require live virus, cell culture, or high-level biosafety laboratories, but instead reflects the ability of antibodies to block the viral receptor binding process through visual or semi-quantitative signals on the paper-based platform. Compared with traditional virus neutralization tests, this method is safer, faster, and more suitable for primary testing and large-scale post-vaccine immune monitoring.

Beyond direct detection of neutralizing antibodies, μPADs have also been used for detection of viral antibodies, antigens, and nucleic acids, which provide an important technical foundation for neutralizing antibody monitoring. Johnson et al. developed automated microfluidic immunoassay methods for SARS-CoV-2 antibody and antigen detection, indicating that microfluidic immune platforms can be used to integrate complex immune reactions and improve detection automation [65]. Yin et al. reported an autonomous lab-on-paper platform for multiplex CRISPR-based SARS-CoV-2 diagnosis; although its primary target is nucleic acid detection, this study reflects the potential of paper-based platforms in multi-step reactions, automated fluid control, and multiplex detection [66]. These technical features can also inform the development of future neutralizing antibody detection platforms.

For variant monitoring, μPADs also have potential advantages. The continuous emergence of SARS-CoV-2 variants makes it difficult for antibody detection with a single antigen or single strain to comprehensively reflect vaccine protection capability. The handheld NIR-to-NIR platform developed by Song et al. can be used to evaluate protective neutralizing antibodies against both the SARS-CoV-2 ancestral strain and Omicron variants in the field after vaccination or infection, illustrating the application value of portable detection platforms in variant immune monitoring [29]. Although this study is not a typical μPADs, its concept of “field-based, portable, multi-strain neutralizing antibody assessment” is highly consistent with the future development direction of μPADs.

In addition, μPADs are also of great significance for blood sample processing. Neutralizing antibodies are usually present in serum or plasma, while field testing prefers to use trace samples such as fingertip blood. Lee et al. reviewed lab-on-paper diagnostic devices based on blood samples, pointing out that paper-based platforms can integrate steps such as plasma separation, sample transport, and detection reactions, thereby reducing the requirements for sample preprocessing [19]. For vaccine-induced neutralizing antibody monitoring, if μPADs can stably handle whole blood or trace blood samples, it would be more beneficial for practical applications in community screening, primary healthcare, and resource-limited areas.

For immune monitoring of other viral diseases, μPADs also have expandable value. Nguyen et al. discussed immune responses, cross-reactions, and vaccine evaluation issues in DENV, JEV, and ZIKV infections [67]. Since there is high antigenic similarity among these flaviviruses, with pronounced antibody cross-reactivity, neutralizing antibody monitoring needs not only to determine whether antibodies are present, but also to distinguish their specificity and cross-reactivity. μPADs can simultaneously detect antibodies related to different viruses or serotypes through multi-channel or multi-detection zone design, and are expected to provide a more flexible platform for vaccine evaluation of dengue, Zika virus, and other viruses.

### 4.3. Advantages and Limitations of μPADs

The main advantages of μPADs lie in low cost, small volume, low sample and reagent consumption, and the ability to complete liquid transport and detection reactions without complex external equipment. Compared with traditional laboratory neutralizing antibody detection methods, μPADs are more suitable for field and large-scale detection. Compared with ordinary LFAs, the structural design of μPADs is more flexible and can complete more complex detection processes through multi-channel, multi-reaction zone, three-dimensional origami structures, and automated fluid control [20,24]. This advantage makes μPADs particularly suitable for semi-quantitative detection, multi-indicator detection, and immunoassays requiring sample preprocessing.

Another advantage of μPADs in neutralizing antibody detection is that they help reduce the technical threshold of traditional neutralization tests. Traditional virus neutralization tests usually require live virus, cell culture, and higher-level laboratories, whereas μPAD-based surrogate neutralization detection can indirectly evaluate antibody function by simulating the virus-receptor binding process [1]. This detection mode retains the core idea of “functional blocking” while reducing biosafety risks and operational complexity. μPADs may therefore become an important platform bridging traditional laboratory neutralization tests and field rapid detection.

μPADs still have clear limitations, however. First, the paper-based materials themselves have batch-to-batch variability, and the pore size, thickness, fiber structure, and hydrophilicity of different papers may affect liquid flow rates and reaction times, leading to insufficient reproducibility of detection results [25]. Second, while the liquid control of μPADs is more flexible than ordinary test strips, it is also more susceptible to the effects of channel size, environmental humidity, evaporation, and sample viscosity. Ardakani and Hemmateenejad systematically summarized the challenges in μPAD development, pointing out that sensitivity, stability, manufacturing consistency, real sample adaptability, and clinical translation remain important issues in this field [25].

In addition, the multiplex detection capability of μPADs has theoretical advantages, but in practical applications may face signal crosstalk, cross-contamination between channels, and inconsistent reaction conditions. For neutralizing antibody detection, this issue is particularly important, as antibody cross-reactivity may exist between different viruses, different variants, or different serotypes. If the antigen selection is not specific enough, or the detection format cannot effectively distinguish neutralizing antibodies from non-neutralizing binding antibodies, results may overestimate the level of protective immunity [67]. When using μPADs for neutralizing antibody detection, attention must therefore be paid to antigen design, detection mechanism, and functional validation.

Furthermore, the results of μPADs still need to be compared and validated with standard neutralization tests or surrogate neutralization tests. Girl et al. evaluated the performance of two rapid LFAs and two surrogate ELISAs in SARS-CoV-2 neutralizing antibody detection, indicating that although rapid detection methods offer application convenience, their performance still needs to be verified through standard methods [68]. This applies equally to μPADs. Future μPADs research should further establish unified calibration standards, optimize signal quantification methods, improve the stability of real sample detection, and conduct more thorough clinical validation in vaccinated populations.

## 5. Paper-Based ELISA for Binding-Antibody and Surrogate Neutralization Assessment

### 5.1. Principle and Detection Modes of Paper-Based ELISA

Paper-based ELISA is an improved form of traditional enzyme-linked immunosorbent assay on paper-based materials. Traditional ELISA uses polystyrene microplates as solid-phase carriers, and achieves detection of target molecules through antigen or antibody immobilization, sample incubation, washing, enzyme-labeled secondary antibody binding, and substrate coloration. Paper-based ELISA replaces the solid-phase carrier with filter paper, nitrocellulose membrane, or other paper-based materials, exploiting the porous fiber structure of paper to immobilize antigens or antibodies and promoting the flow of samples and reagents through capillary action [30,69]. This method retains the antigen–antibody specific recognition and enzyme-catalyzed signal amplification mechanisms of traditional ELISA, while reducing sample volume, reagent consumption, and equipment dependence. When interpreting paper-based ELISA results, conventional antigen-coated formats should be considered primarily as binding antibody assays, whereas competitive or receptor-blocking ELISA configurations are more appropriate for surrogate neutralization readouts. Care should be taken not to describe binding-antibody ELISA signals directly as neutralizing antibody detection.

**Figure 3:**
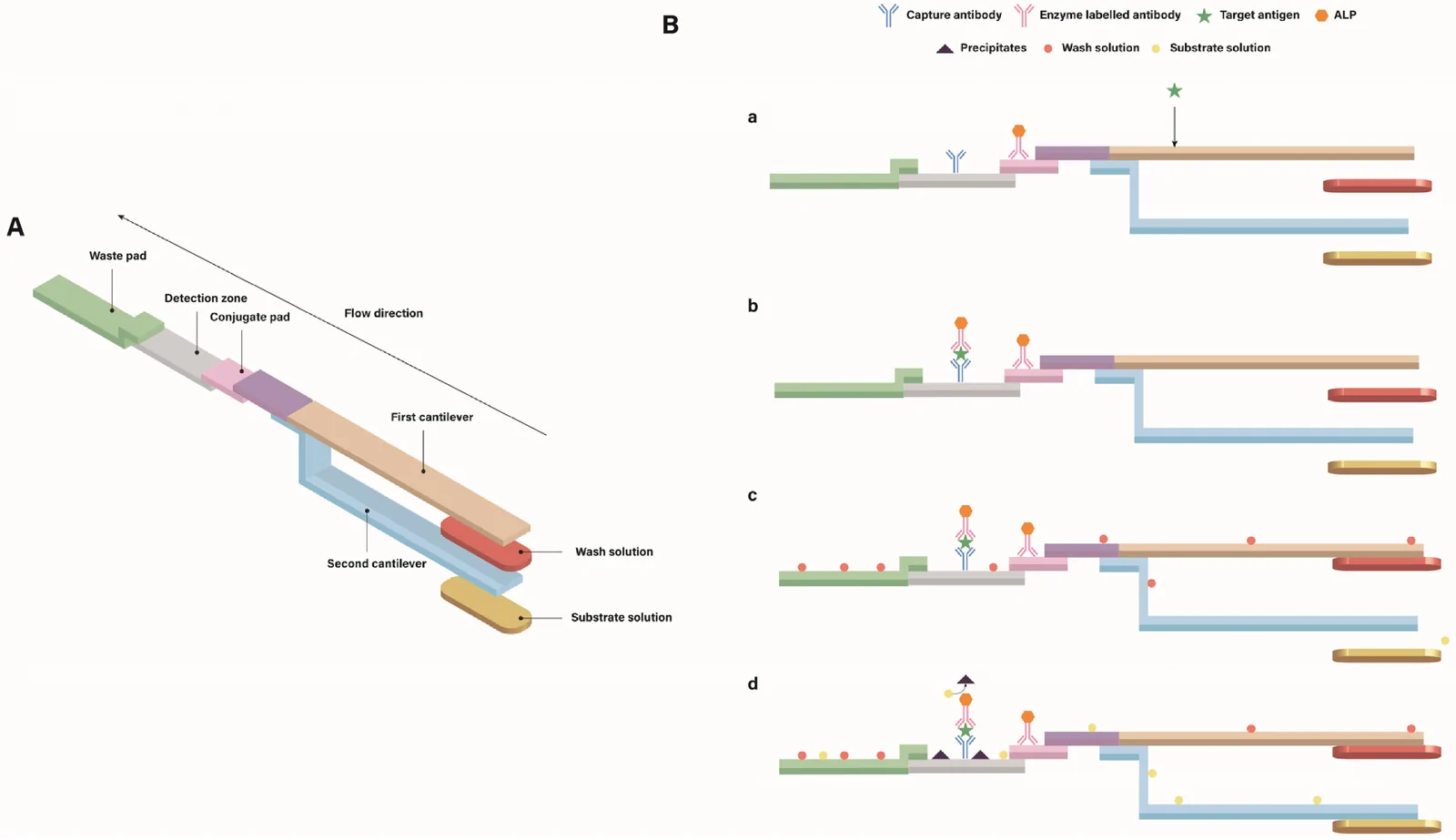
Schematic of the cantilever-controlled paper-based ELISA device. (**A**) Structural layout of the device. (**B**) Stepwise detection principle of the sandwich-type paper-based ELISA: (**a**) sequential sample addition; (**b**) immune complex formation; (**c**) washing; (**d**) enzymatic signal generation at the detection zone [70].

The basic process of paper-based ELISA includes capture molecule immobilization, sample addition, antigen–antibody binding, washing, enzyme-labeled probe binding, and colorimetric or luminescent detection—steps that can be sequentially executed on a single paper-based device through integrated fluidic control, as exemplified by cantilever-driven designs (Figure 3) [70]. Its signal output is most commonly colorimetric, but can also be combined with fluorescence, chemiluminescence, or nanomaterial-enhanced signals. The paper-based multi-well depletion ELISA proposed by Lee et al. designs the paper-based platform into multiple reaction zones, making it similar in structure to a microplate, but with lower cost and smaller size [30]. This design indicates that the reaction logic of traditional ELISA can be transferred to a paper-based platform for building more portable immune detection systems.

Paper-based ELISA can be configured either to detect virus-specific binding antibodies or to estimate receptor-blocking activity through competitive formats. Taking SARS-CoV-2 as an example, RBD, ACE2, or related antigens can be immobilized on a paper-based platform, and the neutralizing ability can be inferred by detecting the degree of inhibition of RBD-ACE2 binding by serum antibodies. This idea is similar to the surrogate virus neutralization test, but paper-based ELISA can further reduce equipment requirements and shorten detection time [1,68]. Paper-based ELISA, therefore, has the potential to become a transitional platform between traditional ELISA, sVNT, and field rapid detection.

The performance of paper-based ELISA depends largely on the method of protein immobilization. Chen et al. compared covalent protein immobilization methods in paper-based ELISA, pointing out that different immobilization strategies affect protein retention capacity, biological activity, and detection signal [69]. For neutralizing antibody detection, the immobilization efficiency of RBD, ACE2, viral antigens, or capture antibodies directly determines detection sensitivity and specificity. If immobilization is not firm, proteins may be lost during the reaction process; if the immobilization method disrupts the protein conformation, it will reduce the ability of antigen–antibody recognition. Protein immobilization is therefore a key technical link for paper-based ELISA to achieve stable application.

### 5.2. Applications of Paper-Based ELISA in Antibody and Surrogate Neutralization Assessment

The number of direct studies of paper-based ELISA in neutralizing antibody detection is fewer than for LFA, but related research has already shown its application potential. The paper-based multi-well depletion ELISA developed by Lee et al. shows that the porous structure of paper can be used to build an immune reaction platform similar to a microplate, and can complete multi-zone detection within a smaller space [30]. This multi-zone structure has potential value for vaccine-induced neutralizing antibody monitoring, as it can simultaneously detect different antigens, different sample dilutions, or different virus strain-related antibodies, thereby obtaining more comprehensive immune response information.

Heidari-Bafroui et al. developed a lab-on-paper microfluidic platform based on a bi-material cantilever actuator for ELISA on paper (Figure 3). As shown in the structural layout (Figure 3A), the device integrates a waste pad, a detection zone pre-coated with capture antibodies, a conjugate pad storing enzyme-labeled antibodies, and two cantilever-actuated reagent reservoirs that sequentially release wash solution and substrate solution upon mechanical actuation. The stepwise operation (Figure 3B) proceeds through sample addition, sandwich immune complex formation, washing to remove unbound reagents, and finally enzymatic signal generation at the detection zone. The significance of this design lies in its ability to achieve automated liquid control through paper-based microfluidic structures and mechanical actuators—making the reagent addition, incubation, washing, and substrate delivery processes more controllable than manual paper-based ELISAs [70]. For neutralizing antibody detection, such automated liquid control is particularly valuable, as competitive or blocking ELISAs impose stringent requirements on incubation timing, washing efficiency, and background suppression.

Paper-based ELISA can also be combined with surrogate ELISA for SARS-CoV-2 neutralizing antibody detection. Girl et al. compared the performance of two rapid LFAs and two surrogate ELISAs for detecting SARS-CoV-2-specific neutralizing antibodies, suggesting that both rapid detection and surrogate ELISA have potential for neutralizing antibody monitoring, but the accuracy and consistency between different methods need systematic evaluation [68]. Tuells et al. used rapid LFIC and sVNT-ELISA to detect SARS-CoV-2 neutralizing antibodies after vaccination in healthcare workers at a large Spanish hospital, indicating that such methods can be used for post-vaccination immune monitoring in real vaccinated populations [71]. Although these studies are not all strictly paper-based ELISA, they provide methodological and application-scenario references for the development of paper-based ELISA for neutralizing antibody detection.

For signal enhancement, nanomaterials can further improve the detection performance of paper-based ELISA. Pang et al. summarized the role of nanomaterials in improving the detection performance of paper-based analytical devices, pointing out that nanomaterials can improve detection sensitivity and stability by enhancing optical signals, catalytic reactions, or electrochemical responses [31]. Zhu et al. developed TimePAD, using deep learning to parse ELISA time-series signals in microfluidic paper-based platforms to achieve rapid readout and improve accuracy [72]. This study indicates that paper-based ELISA can be combined with AI and image analysis to overcome the problems of subjective readout and insufficient quantification in traditional paper-based detection.

The application of paper-based ELISA in vaccine-induced neutralizing antibody monitoring can therefore be summarized in three directions: first, miniaturizing and reducing the cost of traditional ELISA for antibody level detection; second, introducing the blocking reaction of sVNT into a paper-based platform for functional neutralizing antibody evaluation; third, combining nanomaterials, microfluidic structures, and intelligent readout technology to improve sensitivity, automation, and quantitative capability.

### 5.3. Advantages and Limitations of Paper-Based ELISA

The main advantage of paper-based ELISA lies in combining the immunological specificity of traditional ELISA with the low cost and portability of paper-based platforms. Compared with traditional microplate ELISA, paper-based ELISA requires less sample and reagent volume, shorter detection time, and lower equipment dependence [30]. These characteristics make it more suitable for primary healthcare, vaccination sites, resource-limited areas, and large-scale serological surveys. For vaccine-induced neutralizing antibody monitoring, paper-based ELISA can serve as a low-cost, relatively easy-to-quantify immunoassay platform for assessing antibody-level changes after vaccination.

Compared with ordinary LFA, the potential advantage of paper-based ELISA is that its enzyme-catalyzed signal amplification capability is stronger, theoretically making it more suitable for semi-quantitative or quantitative analysis. LFA relies on the color intensity of detection lines to determine results, which is easily affected by naked eye observation, lighting conditions, and sample flow rate; paper-based ELISA can obtain more stable signals through enzyme-catalyzed coloration, image analysis, or portable reading devices [30,72]. In scenarios that require comparison of antibody levels at different time points, evaluation of booster vaccination effects, or population immune monitoring, paper-based ELISA may therefore have greater quantitative potential than simple LFA.

Paper-based ELISA also has obvious shortcomings, however. First, protein immobilization remains a key challenge. Chen et al.’s study showed that different covalent immobilization methods affect the stability of proteins on paper and detection effects [69]. For neutralizing antibody detection, if RBD, ACE2, or antibodies are not stably immobilized on paper, it may lead to reduced signal, worse reproducibility, or elevated background. Second, the washing process of paper-based ELISA is more difficult to precisely control than traditional microplate ELISA. If too many unbound components remain, higher background signals result; if washing is too intense, loss of target complexes may occur, thereby affecting sensitivity and quantitative accuracy [70].

In addition, the operational steps of paper-based ELISA are still more than those of ordinary LFA. Although more portable than traditional ELISA, most paper-based ELISAs still require steps such as sample addition, incubation, washing, addition of enzyme-labeled probes, and addition of substrate. This multi-step process may increase human error and is not conducive to truly home-based self-testing or operation by non-professionals. Future paper-based ELISA therefore needs to further develop pre-stored dry reagents, automated liquid control, and one-step or few-step detection processes [70,72].

Finally, paper-based ELISA for neutralizing antibody detection still needs more real samples and clinical cohort validation. The key to neutralizing antibody detection is to reflect protective immune function, not simply detecting binding antibodies. If paper-based ELISA only detects antigen-binding antibodies, it may not fully represent neutralization capability. Future research should therefore more frequently adopt competitive or blocking designs, and compare with PRNT, pVNT, sVNT, or other standard methods [68,71]. Only after systematic validation can paper-based ELISA be more reliably used for post-vaccine immune monitoring and public health decision-making.

Overall, paper-based ELISA forms an important bridge between traditional laboratory ELISA and field rapid detection. It retains the immune recognition and signal amplification advantages of ELISA while possessing the low-cost, miniaturized, and portable characteristics of paper-based platforms. With the development of protein immobilization technology, automated microfluidic control, nanomaterial enhancement, and AI readout technology, paper-based ELISA is expected to become an important complementary platform for vaccine-induced neutralizing antibody monitoring.

## 6. Applications Across Viral Diseases

### 6.1. SARS-CoV-2

SARS-CoV-2 is the most extensively studied viral target for paper-based antibody monitoring. Protective humoral immunity is largely directed against the Spike glycoprotein, particularly the receptor-binding domain (RBD), because many neutralizing antibodies interfere with RBD engagement of angiotensin-converting enzyme 2 (ACE2). This molecular interaction has enabled the development of paper-based assays that either detect Spike- or RBD-binding antibodies or estimate receptor-blocking activity through competitive RBD–ACE2 formats. These two assay categories should be distinguished: antigen-binding signals indicate virus-specific humoral responses, whereas competitive inhibition provides a surrogate, but not direct, measure of functional neutralization [2,3,6,10,44,73]. Across LFA, μPAD, and paper-based ELISA formats, SARS-CoV-2 assays vary substantially in their biological meaning. RBD-coated strips or paper-based ELISAs that capture anti-RBD IgG or IgM should be classified as binding-antibody assays, even when their signals correlate with neutralization in a particular cohort. By contrast, formats in which sample antibodies reduce RBD–ACE2 binding can be classified as receptor-blocking or surrogate neutralization assays. Digital imaging, fluorescence, and smartphone-assisted analysis can improve quantification, but these readout technologies do not by themselves establish functional relevance. For longitudinal vaccine monitoring, assay outputs should therefore be calibrated against pVNT, VNT/PRNT, or a validated sVNT and should report the relevant variant, sample dilution, cutoff definition, and reference method [1,7,8,10,68,72,74,75,76,77,78].

In terms of detection platforms, SARS-CoV-2 paper-based antibody detection includes not only qualitative or semi-quantitative LFAs, but also paper-based enzyme-linked immunosorbent assay (paper-based ELISA) and microfluidic paper-based analytical devices (μPADs). LFA is rapid, low-cost, easy to operate, and suitable for field use, making it useful for large-scale serological screening and point-of-care testing. Paper-based ELISA combines the high specificity and sensitivity of conventional ELISA with the portability and low reagent consumption of paper-based platforms, and can be used to detect SARS-CoV-2-related IgG and IgM antibodies. μPADs enable more complex sample processing, reaction control, and quantitative analysis through microfluidic channels and multi-reaction-zone designs. Together, these platforms demonstrate the diverse application potential of paper-based materials in SARS-CoV-2 antibody detection [1,17,18,65,79].

Beyond qualitative detection, quantitative detection is particularly important for SARS-CoV-2 vaccine-induced immune monitoring. Post-vaccination antibody levels change over time, and simply classifying antibodies as positive or negative is insufficient to reflect the dynamic changes in immune protection. Platforms such as paper-based ELISA and μPADs are therefore used for quantitative detection of IgG and IgM antibody levels against SARS-CoV-2 to assess the kinetics of vaccine-induced antibody responses. Related studies show that antibody levels measured by paper-based detection platforms have a strong correlation with neutralizing activity determined by pseudovirus neutralization tests, with relatively high correlation coefficients in some studies, indicating that paper-based binding-antibody immunoassays indirect correlates of neutralization or as complementary monitoring tools when their association with reference neutralization methods has been demonstrated. Since conventional neutralization tests are often time-consuming, labor-intensive, and may require higher biosafety-level conditions, the emergence of paper-based platforms provides a more convenient technical approach for nAb monitoring [8,9].

For clinical and epidemiological applications, SARS-CoV-2 paper-based biosensors have multiple uses. First, they can be used for dynamic monitoring of antibody titers after vaccination, helping to understand the duration of humoral immune responses. Longitudinal studies show that nAb levels after mRNA vaccination peak within a few weeks and then gradually decline, with differences between individuals, possibly influenced by factors such as vaccine type, age, and previous infection status. Regular antibody monitoring through paper-based platforms therefore helps determine whether immune protection has weakened, providing a basis for the timing of booster vaccination [13,14].

Second, paper-based detection can be used to assess the risk of breakthrough infections. Over time or with the emergence of viral variants, nAb levels in some individuals may decline, increasing the risk of infection. By rapidly identifying populations with low or rapidly declining nAb levels, paper-based detection can support targeted public health interventions. For example, in nursing homes, immunocompromised populations, healthcare workers, and other high-risk groups, rapid antibody monitoring can help determine whether booster vaccination prioritization or additional protective measures are needed [9,15,16].

Third, paper-based detection also has practical value in convalescent plasma therapy and passive immunization strategies. The therapeutic effect of convalescent plasma is closely related to the nAb titer in the donor plasma, so it is necessary to rapidly screen plasma donors with high nAb titers. Compared with conventional neutralization tests, paper-based detection can complete initial screening in a shorter time, improving the efficiency of donor screening and providing a more timely basis for passive immunization strategies [80].

In addition, the development of SARS-CoV-2 paper-based nAb detection is also consistent with the urgent needs of resource-limited areas for accessible, rapid, and accurate immune assessment tools. Paper-based platforms do not rely on large instruments, are easy to operate, and can be used outside central laboratories, thereby expanding the coverage of immune monitoring. Their portability and ease of use make them suitable for applications in primary healthcare institutions, community testing sites, vaccination sites, and resource-limited areas. Compared with traditional laboratory testing, paper-based testing is more conducive to decentralized and large-scale population immune monitoring [10,12].

The continuing evolution of SARS-CoV-2 limits the transferability of a fixed-antigen assay. Mutations within the RBD and other Spike epitopes may reduce recognition by antibodies induced by earlier infection or vaccination, so a strong signal against an ancestral antigen does not necessarily indicate equivalent activity against a newly circulating variant. Variant-matched antigens, multivariant test panels, and periodic recalibration against functional neutralization data are therefore important for maintaining clinical interpretability. At present, paper-based SARS-CoV-2 assays are most defensible as rapid tools for serological screening, longitudinal trend monitoring, and decentralized surveillance; claims regarding individual protection or booster decisions require substantially stronger clinical validation [4,5,9,15,29].

### 6.2. Influenza

Influenza virus presents a different assay-design problem because protective antibody responses are strongly strain dependent. Hemagglutinin (HA) is the principal target of classical neutralizing antibodies: antibodies directed to the HA receptor-binding head can block attachment, whereas antibodies against conserved stem epitopes may provide broader but often less abundant activity. Neuraminidase (NA)-specific antibodies can also contribute to protection by limiting viral release and spread. Consequently, HA- or NA-binding signals should be interpreted as antigen-specific antibody responses unless a blocking or functional relationship has been independently demonstrated [81,82].

Paper-based LFAs, μPADs, and paper-based ELISAs can support rapid measurement of vaccine-induced influenza antibodies, but antigenic drift, subtype diversity, repeated vaccination, and prior infection complicate the relationship between signal intensity and protection. An assay constructed with a single historical HA antigen may not adequately represent immunity to the currently circulating strain. Where practical, strain-matched antigens or multiplex panels containing representative HA and NA antigens should be used, and performance should be compared with hemagglutination-inhibition, microneutralization, or other accepted functional methods [81,82,83].

The potential value of paper-based platforms in influenza antibody monitoring is mainly reflected in three aspects. First, lateral flow assays (LFAs) can use recombinant HA, NA, or virus-like particles as detection antigens to rapidly screen for vaccine-induced specific antibodies. Second, microfluidic paper-based analytical devices (μPADs) can simultaneously detect antibodies associated with different influenza subtypes, such as H1N1 and H3N2, through multi-channel or multi-detection-zone designs, thereby assessing the breadth of vaccine-induced antibody responses. Third, paper-based platforms combined with smartphone readouts, fluorescent probes, or nanomaterial-enhanced signals are expected to improve quantitative antibody detection, making them suitable for population immune monitoring after seasonal influenza vaccination. Moreno et al.’s clinical study on yeast β-glucan enhancement of influenza vaccine antibody responses also indicates that vaccine-induced antibody levels are affected by immune enhancers, individual conditions, and vaccination strategies; therefore, simple tools are needed for dynamic monitoring [84].

Influenza paper-based nAb detection also has important public health significance. Since the influenza virus epidemic strain may change each year, if antibody levels in populations in communities, schools, nursing homes, or primary healthcare institutions can be rapidly assessed, it helps identify populations with insufficient immunity, guiding booster vaccination and vaccine strain selection. Svyatchenko et al. analyzed the seroprevalence of anti-influenza antibodies in the 2023–2024 influenza season and the characteristics of seasonal strains, indicating that influenza antibody monitoring has practical significance for understanding the immune background and virus transmission during epidemic seasons [83]. Compared with traditional laboratory methods, paper-based detection platforms have advantages in detection speed, low sample requirements, simple operation, and ease of on-site deployment, and are suitable for large-scale serological surveys and preliminary vaccine efficacy screening.

At present, however, research on paper-based assays specifically targeting influenza nAbs remains less extensive than that on SARS-CoV-2. Existing studies focus more on influenza antigen detection, binding antibody detection, or conventional serological evaluation, and evidence directly linking paper-based platforms to functional nAb detection remains limited. When discussing influenza in a review, it is therefore important to avoid equating all influenza antibody detection with nAb detection. A more rigorous formulation is that paper-based platforms have application potential in post-influenza-vaccine antibody screening and field-based immune monitoring, but their results still need to be compared and validated against functional assays such as HAI tests and microneutralization assays. In addition, Alam et al.’s study on potential cross-reactivity between SARS-CoV-2-specific nAbs and influenza A virus suggests that complex cross-immunity phenomena may occur between different respiratory viruses, which also requires future paper-based platforms to further improve specificity in antigen selection and result interpretation [85].

Accordingly, the principal translational opportunity for paper-based influenza antibody testing is not to replace strain-specific neutralization assays, but to provide rapid, decentralized screening of antibody patterns and population-level immune trends. Clinical interpretation should identify the tested strain or subtype and avoid assigning a universal protective meaning to a positive binding-antibody result.

### 6.3. Dengue

Dengue virus (DENV) is a particularly demanding target for paper-based antibody monitoring because four serotypes circulate and antibodies may be serotype specific, cross-neutralizing, non-neutralizing, or associated with antibody-dependent enhancement. The envelope (E) protein contains major neutralization-related epitopes, including epitopes within domain III (EDIII) and quaternary structures formed on the virion surface. However, a binding signal against E protein cannot by itself distinguish protective neutralization from cross-reactive recognition [67,86,87].

In dengue vaccine evaluation, nAb levels are an important indicator of protective immunity, but conventional methods such as PRNT are labor-intensive, time-consuming, and require specialized laboratory facilities, limiting their use for large-scale field monitoring. Paper-based biosensors provide a low-cost, rapid, and portable alternative for post-vaccination antibody screening. In principle, LFAs and μPADs can incorporate envelope proteins, EDIII, or other serotype-specific antigens from the four DENV serotypes into separate detection zones, enabling multiplex assessment of vaccine-induced antibody responses and supporting seroepidemiological surveillance in dengue-endemic regions. However, this approach faces a major challenge in regions where DENV, ZIKV, Japanese encephalitis virus, and other flaviviruses co-circulate. Conserved flavivirus epitopes can generate broadly reactive or false-positive signals, causing paper-based binding assays to overestimate virus-specific immunity or misclassify the infecting serotype. Consequently, antigen selection and assay design are critical determinants of analytical specificity and clinical interpretation, directly influencing serosurveillance, vaccine evaluation, and assessment of individual immune status [67,86,87,88].

The biggest challenge in DENV detection is cross-reactivity. DENV, Zika virus (ZIKV), Japanese encephalitis virus (JEV), and other flaviviruses share high structural similarity in envelope proteins, and traditional serological methods are prone to false positives or difficulty distinguishing the type of previous infection. Nguyen et al. systematically discussed the immune responses in DENV, JEV, and ZIKV infections and their effects on cross-reactivity and vaccine evaluation, pointing out that antibody cross-recognition among flaviviruses substantially increases the difficulty of serological interpretation [67]. This is particularly important for paper-based detection platforms, as many paper-based immunoassays essentially detect antigen–antibody binding, and binding antibodies do not necessarily have neutralizing activity. If the antigen design is not specific enough, paper-based platforms may mistakenly identify non-neutralizing cross-antibodies as protective antibodies.

Future paper-based platforms for DENV nAb monitoring should prioritize more precise antigen selection, particularly serotype-discriminating envelope protein domains or epitope combinations rather than relying exclusively on the whole E protein. EDIII is a promising candidate because it contains receptor-binding and neutralization-associated epitopes and may reduce interference from cross-reactive non-neutralizing antibodies compared with more conserved antigenic regions. Although EDIII-focused strategies have been more extensively explored in ZIKV and broader flavivirus vaccine research, the principle of using more specific antigenic targets to improve nAb detection accuracy is equally applicable to DENV [89,90]. Multiplex platforms incorporating antigens from all four DENV serotypes together with ZIKV- and JEV-related antigens may further help distinguish antibody origin and cross-reactivity patterns, while competitive formats and orthogonal confirmation assays can improve analytical discrimination. However, clinically meaningful interpretation still requires correlation with serotype-specific PRNT or another appropriate functional reference method. Because DENV lacks a single receptor-blocking interaction that can be reproduced as readily as the SARS-CoV-2 RBD–ACE2 system, most current paper-based approaches should be described as binding-antibody assays or indirect neutralization-related assays rather than direct measures of functional neutralization.

Paper-based platforms also have strong public health value in DENV applications. DENV mainly circulates in tropical and subtropical regions, many of which have limited medical resources, making it difficult for traditional laboratory testing to cover primary-level communities. Khor et al. conducted a survey on the seroprevalence and related factors of chikungunya, dengue, Japanese encephalitis, and Zika in forest-edge residents, suggesting that in areas where multiple arboviruses co-circulate, there is an important need for rapid, low-cost, and field-based antibody detection tools [88]. If paper-based detection platforms can differentiate between DENV serotype-specific antibodies and cross-reactive antibodies, it would assist in pre-vaccination screening, post-vaccination immune monitoring, and epidemiological risk assessment.

DENV paper-based nAb detection is still in the development stage; however, it cannot be simply equated with the relatively mature RBD-ACE2 blocking detection in the SARS-CoV-2 field. DENV does not have a single, clear, and easily simulated in vitro receptor-blocking system like SARS-CoV-2 RBD-ACE2, making it more difficult for paper-based platforms to achieve truly functional nAb detection. Currently, a more realistic strategy is to use paper-based platforms for serotype-specific binding antibody screening, combined with multiplex antigen design, competitive detection modes, and standard neutralization test validation, to improve the predictive value for protective immunity.

The most realistic near-term role of paper-based DENV platforms is decentralized serological screening and population surveillance, particularly in resource-limited endemic settings. Their development should focus on multiplex serotype discrimination, geographically relevant cross-reactivity panels, and validation using samples with known infection, vaccination, and neutralization profiles.

### 6.4. Zika

Zika virus (ZIKV) antibody monitoring faces many of the same flavivirus cross-reactivity problems as DENV, but the clinical consequences of misclassification are distinct because ZIKV infection is associated with congenital and neurological disease. Neutralization-related antibodies mainly recognize epitopes on the E protein, including EDIII and other conformational regions involved in attachment and entry. Epitope-focused antigens may improve analytical specificity, but no single binding antigen completely resolves DENV–ZIKV discrimination in all exposure settings [67,89,90].

The key issue in ZIKV detection is antigen selection. Due to the high homology of flavivirus envelope proteins, antibodies targeting conserved epitopes tend to cross-react, making it difficult for traditional serological detection to distinguish ZIKV and DENV infections. Georgiev et al. developed resurfaced ZIKV EDIII nanoparticle immunogens that can induce in vivo neutralizing and protective responses, suggesting that EDIII is an important target for improving the specificity and accuracy of ZIKV immune detection and nAb detection [89]. EDIII contains relatively more virus-specific epitopes; if used as the capture antigen in paper-based LFAs or μPADs, it may reduce cross-reactivity with DENV and other related flavivirus antibodies, improving detection specificity.

Vaccine research also suggests that ZIKV antibody monitoring needs to balance protection and safety. Liu et al. reported a single-dose circular RNA ZIKV vaccine that can prevent ZIKV infection and does not exacerbate dengue severity [90]. This has important implications for detection platform design: ZIKV vaccine evaluation should not only assess whether high antibody levels have been induced, but must also focus on whether these antibodies have neutralizing function, and whether they may have adverse cross-reactive effects with DENV. Future paper-based ZIKV antibody detection platforms should therefore prioritize developing detection systems that can identify ZIKV-specific neutralizing epitopes, and minimize detection of large quantities of non-neutralizing cross-reactive antibodies.

Multiplex detection is an important direction for the development of paper-based ZIKV platforms. Merbah et al. established a high-throughput multiplex assay for characterizing flavivirus-specific immunoglobulins, demonstrating that more comprehensive differentiation of different flavivirus infections or immune responses can be achieved through multi-antigen, multi-marker detection [86]. Although this study is not a paper-based platform, its multiplex immunoassay approach can provide references for the design of paper-based μPADs and multi-zone LFAs. Future paper-based platforms can arrange ZIKV EDIII, different DENV serotype antigens, and JEV-related antigens in the same device, and determine the origin and degree of cross-reactivity of antibodies based on the signal patterns of different detection zones. This approach is particularly suitable for vaccine evaluation and population immune monitoring in areas where ZIKV and DENV co-circulate.

For paper-based testing, multiplex DENV–ZIKV antigen panels are likely to be more informative than single-analyte formats in co-circulation regions. Competitive designs, paired antigen domains, and orthogonal confirmation may further reduce misclassification. Validation cohorts should include individuals with prior DENV infection or vaccination, confirmed ZIKV infection, other flavivirus exposures, and samples characterized by functional neutralization methods.

Currently, however, there is insufficient evidence for ZIKV paper-based nAb detection. Existing literature focuses more on ZIKV vaccine immunogen design, cross-reactivity mechanisms, and multiplex serological analysis, with limited research that actually applies paper-based platforms to ZIKV functional nAb detection. In a review, the ZIKV section should therefore be positioned as “an application direction with important needs and development potential” rather than a mature application area. Future research needs to combine ZIKV-specific antigen design, multiplex paper-based detection, digital readouts, and standard neutralization test validation to improve the interpretive capability of detection results for protective immunity.

Overall, the key to ZIKV paper-based nAb detection lies in solving the problems of flavivirus cross-reactivity and functional validation. Compared with single antibody positivity testing, a more valuable future direction is to construct multiplex paper-based detection platforms that can simultaneously distinguish antibody responses to ZIKV, DENV, and other flaviviruses, and to clarify their actual application value in vaccine evaluation and public health surveillance through correlation analysis with PRNT or other functional neutralization tests. Because direct evidence for paper-based functional ZIKV neutralization testing remains limited, this application should be presented as an emerging direction rather than a mature clinical use. At present, the strongest use cases are field screening, seroepidemiological surveillance, and research-oriented vaccine-response monitoring, with individual protection claims reserved for assays supported by functional validation.

### 6.5. Monkeypox

The recent global spread of monkeypox virus (MPXV) has increased the demand for monitoring vaccine-induced immune responses. Since 2022, MPXV outbreaks have spread to multiple non-endemic regions, prompting public health authorities to focus increasingly on population-level protective immunity, vaccine effectiveness, and booster immunization strategies. Modified Vaccinia Ankara-Bavarian Nordic (MVA-BN), a third-generation smallpox vaccine, has been widely used for MPXV prevention because of its favorable safety profile and immunogenicity against MPXV. However, the magnitude, durability, and population-level variability of MPXV-specific nAbs after MVA-BN vaccination still require further clarification. Establishing rapid, accessible, and field-deployable antibody monitoring tools is therefore important for evaluating vaccine-induced protective immunity [58].

The MPXV A29L protein is an important target in current paper-based antibody detection research. A29L is a homolog of vaccinia virus A27L and has an important role in viral adsorption and entry into host cells; it is therefore not only a potential antigen in vaccine design but is also suitable as a target for serological diagnosis and antibody monitoring. Based on this characteristic, researchers have recently developed paper-based LFA methods for MPXV A29L-specific antibodies. Such LFAs offer rapid, low-cost, simple operation, and suitability for point-of-care testing, and can be used for humoral immune monitoring in different populations [59].

In terms of detection technology, paper-based LFAs targeting MPXV A29L can capture antibody responses in samples through specific antigens or monoclonal antibodies, and use colloidal gold, fluorescent quantum dots, or other nanomaterials to generate readable signals. Dual-signal LFAs have been developed using multilayer SiO2-Au core structures combined with dual quantum dot nanocomposites to achieve ultrasensitive detection of MPXV A29L antigen. This detection can be completed in about 15 min, with a low detection limit in fluorescence mode, and sensitivity markedly better than traditional ELISA. Although this study mainly shows the antigen detection capability for A29L, its platform design and signal enhancement strategy also provide an important technical foundation for MPXV-specific antibody and vaccine-induced immune monitoring [60].

Preliminary research indicates that paper-based biosensors targeting MPXV-specific antibodies have strong potential for distinguishing vaccinated from unvaccinated individuals. These detection devices can capture and detect specific immunoglobulins generated after vaccination or natural infection using monoclonal antibodies or recombinant antigens against A29L. Some monoclonal antibodies have been shown to specifically recognize MPXV A29L without cross-reacting with vaccinia virus A27L, thereby improving the specificity of LFA detection. This is particularly important for MPXV serological detection, as there is some antigenic similarity among orthopoxviruses, and if the detection target is not selected carefully, cross-reactivity may occur and affect result interpretation [59].

Compared with traditional ELISA, MPXV antibody detection based on paper-based LFA has shorter detection time and stronger field applicability. Comparative studies show that the sensitivity and specificity of relevant paper-based detection can be comparable to conventional ELISA methods, but with markedly shorter detection time, usually about 15 min to obtain results. The rapid turnaround makes it particularly suitable for outbreak scenarios, as during an outbreak, public health authorities need to rapidly assess individual and population immune levels to determine whether vaccine coverage is sufficient, whether key populations need booster immunization, and whether prevention strategies need adjustment [60].

The application of fluorescent labels such as quantum dots further enhances the detection performance of MPXV paper-based LFA. Compared with traditional colorimetric labels, quantum-dot fluorescence provides stronger signal intensity and better stability, which can improve detection accuracy and sensitivity. Through portable fluorescence reading devices or other digital readout methods, paper-based LFAs are expected to develop from simple qualitative judgment to semi-quantitative or quantitative antibody monitoring tools. This is significant for assessing differences in antibody levels after MVA-BN vaccination, tracking the process of antibody decay, and determining the need for booster immunization [60].

From a public health perspective, paper-based LFAs can provide important support for rapid population immune assessment during MPXV outbreaks. By conducting field testing in communities, vaccination sites, or primary healthcare institutions, populations with low antibody levels, potentially insufficient immune protection, or inadequate vaccine coverage can be rapidly identified, thereby guiding vaccine distribution and booster vaccination priority. In areas with limited vaccine resources, this rapid screening capability is particularly important, as it can help public health authorities prioritize vaccines for populations with lower immune levels or higher infection risks [91].

Vaccine-induced antibody levels against MPXV may decline over time, further underscoring the necessity of dynamic monitoring. Evidence suggests that vaccine-induced nAb titers decay over time, and some individuals may show low or undetectable antibody responses more than two years after vaccination. A single vaccination record therefore cannot fully represent the current protective immune status. Continuous monitoring through paper-based antibody detection helps determine whether immune waning exists in the population and provides a basis for booster immunization strategies [58,91,92].

In addition, paper-based antibody detection can also be used to assess vaccine effectiveness in the context of emerging MPXV variants. If viral antigens change, the neutralizing ability of existing vaccine-induced antibodies may decline, so tools are needed to rapidly detect antibody responses related to variant strains. Paper-based platforms are modular and can adapt to monitoring needs for different MPXV strains or antigenic variants by replacing antigens or optimizing detection formats. Meanwhile, for immunocompromised populations, such as people living with HIV, rapid antibody detection can help evaluate whether their immune responses after vaccination are sufficient and guide individualized protection and booster immunization strategies [93].

Paper-based detection targeting MPXV-specific antibodies is still in the development and validation stage, but it has shown strong application prospects. LFAs targeting A29L provide a feasible technical route for MPXV vaccine-induced antibody monitoring, and their rapid turnaround, low cost, portability, and field-deployable characteristics can compensate for the shortcomings of conventional laboratory serological testing in epidemic emergencies. Further clinical sample validation is still needed to clarify the correlation between detection results and functional nAb levels, and to evaluate detection performance in populations with different vaccination statuses, exposure backgrounds, and immune statuses. With advances in antigen design, monoclonal antibody screening, fluorescent nanomaterials, and portable readout technology, paper-based MPXV antibody detection may be integrated into epidemic response systems, improving the timeliness and precision of public health interventions and supporting MPXV transmission control and vaccine strategy optimization [58,59,60,91,92,93]. Across the viral targets covered in this section, the key neutralization-related antigens and their implications for paper-based assay design are summarized in Table 3.

## 7. Comparative Analysis of Detection Methods

### 7.1. Overview of Conventional Neutralizing Antibody Detection Methods

Neutralizing antibody (nAb) detection is an important means for evaluating vaccine-induced protective immunity [2,13]. Conventional virus neutralization tests, especially the plaque reduction neutralization test (PRNT) and the microneutralization assay, have long been regarded as gold standards for evaluating the functional activity of nAbs. These methods directly reflect the neutralizing capacity of nAbs by observing the inhibitory effect of nAbs on the ability of viruses to infect cells, and therefore hold relatively high authority in vaccine efficacy evaluation, determination of immune protection levels, and virological research [44,57].

PRNT can provide relatively stable and continuous quantitative results, such as IC50, IC90, or neutralizing titers, used to indicate the concentration or dilution required for antibodies to inhibit 50% or 90% of viral infection [44,56,57]. This quantitative capability helps to more accurately assess the immune protection level of individuals or populations. Conventional neutralization tests require live virus, sensitive cell lines, strict cell culture conditions, and BSL-2 or higher-level laboratory facilities, however. The detection process includes multiple steps such as sample dilution, virus incubation, cell infection, culture observation, and result interpretation, with an overall time requirement of 2–5 days, and relatively high demands on the professional capabilities of operators [3,44,56].

Beyond PRNT and microneutralization assays, the traditional laboratory detection system also includes the surrogate virus neutralization test (sVNT), conventional ELISA, chemiluminescence immunoassay, and flow cytometry methods. Compared with live-virus neutralization tests, some surrogate assays reduce biosafety risks and improve detection throughput, but still rely on centralized laboratories, specialized equipment, and standardized operating procedures. While conventional methods have advantages in accuracy, functional evaluation, and standardization, they therefore have clear limitations in rapid detection, field deployment, and large-scale population monitoring [3,68,94,95].

### 7.2. Comparison of Analytical Performance

In terms of analytical performance, traditional neutralization tests and paper-based biosensors differ in their respective strengths. The core advantage of traditional neutralization tests lies in their ability to directly evaluate whether antibodies have the functional activity of inhibiting viral infection, and therefore their specificity and biological relevance are relatively high [44,57]. PRNT and microneutralization assays can provide continuous quantitative data, helping to compare differences in immune responses between different vaccines, different vaccination time points, or different populations. This is of great importance for vaccine protective efficacy evaluation, immune persistence research, and booster immunization strategy formulation [4,5,9,13,14,15].

Paper-based biosensors, by contrast, mainly include LFA, μPADs, and paper-based ELISA [11,17,20,30]. These platforms are based on antigen–antibody binding reactions and generate signals through colloidal gold, fluorescent labels, enzyme-catalyzed colorimetric reactions, or other signal amplification systems [21,22,31,32]. LFA is the most widely used paper-based platform and can obtain results within 15–30 min [10,75]; μPADs achieve autonomous fluid flow and multichannel reactions of samples and reagents through microchannels on paper, further simplifying the detection process [17,20,96]; paper-based ELISA retains the basic principles of immunoassay while shortening the reaction time, completing detection within about 30–60 min [30,97].

Paper-based methods are still weaker than traditional neutralization tests in quantitative capability, however. Most LFAs primarily provide qualitative or semi-quantitative results, such as positive/negative determinations or test strip color intensity grading [28,75], and cannot provide precise IC50, IC90, or neutralizing titers like PRNT [44,57,76]. In research scenarios requiring precise antibody titer assessment, determination of protection thresholds, or comparison of immune persistence, paper-based detection therefore still needs to rely on conventional methods for validation.

In terms of specificity, traditional neutralization tests directly measure the inhibitory effect of nAbs on viral infection and can better distinguish functional nAbs from non-neutralizing binding antibodies [44,57]. In contrast, some paper-based sensors primarily detect binding antibodies against the viral receptor-binding domain (RBD) or other viral proteins, and binding antibodies do not necessarily have neutralizing activity. If paper-based detection lacks competitive or blocking design, non-nAbs may be mistakenly identified as antibodies with protective significance, thereby affecting the interpretation of immune status [7,8,9,68].

Traditional neutralization tests have advantages in functional evaluation, quantitative accuracy, and result credibility; paper-based biosensors have more potential in rapid response, simplified operation, and expanding detection coverage. The two are not a simple replacement relationship, but are respectively suited for different application levels such as confirmatory testing and field screening. A performance-oriented comparison of these paper-based platforms for vaccine-induced nAb monitoring is presented in Table 4.

### 7.3. Operational and Practical Considerations

In terms of operational convenience and detection speed, paper-based biosensors are markedly superior to traditional neutralization tests [10,12,17]. Conventional methods require live-virus culture, cell operations, long incubation periods, and interpretation by trained personnel, with detection cycles of several days, making them unsuitable for rapid field testing and high-frequency monitoring [44,56]. In contrast, LFAs can obtain results within 15–30 min, and μPADs and paper-based ELISA can also complete detection in a shorter time, making them more suitable for point-of-care testing (POCT) settings.

Paper-based platforms do not require live virus and cell culture, substantially reducing biosafety requirements [10,12,17]. Their detection process mainly relies on capillary action to drive sample and reagent flow, without the need for external pumps, power sources, or complex instruments [17,20,98]. For primary healthcare institutions, community clinics, border checkpoints, vaccination sites, and resource-limited areas, lower equipment dependence is of great practical significance. Healthcare workers with simple training, or even some non-professionals, can complete sample loading, result reading, and preliminary interpretation, thereby expanding the accessibility of nAb detection services [11,17,99].

In terms of cost and accessibility, the overall cost of traditional neutralization tests is relatively high, with a single test may cost tens to hundreds of dollars, more suitable for centralized laboratories, research institutions, or vaccine clinical trial centers, and difficult to routinely apply in large-scale population screening [3,44,56]. Paper-based biosensors have clear cost advantages. Paper substrates are inexpensive, and manufacturing can be achieved through scalable production methods such as wax printing, inkjet printing, 3D printing, or hydrophobic patterning [20,24,25,26]. At the same time, low-cost signal labels such as colloidal gold, enzyme-catalyzed colorimetric systems, fluorescent probes, and nanomaterials can also help improve detection sensitivity without markedly increasing equipment costs [31,32,100]. The per-test cost of paper-based assays can be reduced to $1–5 or even lower, suitable for deployment in resource-limited areas and low- and middle-income countries [11,20,101,102].

In addition, paper-based devices are small, lightweight, easy to transport and store, and single-use disposable, reducing issues such as sample transportation, biohazard waste disposal, and centralized laboratory burden [11,19,20]. When combined with smartphone readout and image analysis algorithms, paper-based platforms can also improve result recording, remote transmission, and data integration. These characteristics give paper-based platforms relatively strong feasibility in decentralized detection and public health field applications [97,103].

### 7.4. Suitability for Vaccine Monitoring and Population Surveillance

In vaccine immune monitoring, traditional neutralization tests and paper-based biosensors have complementary values. Conventional neutralization tests are suitable for vaccine development, clinical trials, immune protection mechanism research, and standard method validation, and can provide highly specific, highly credible functional neutralization data. For determining protective antibody thresholds, comparing the efficacy of different vaccine platforms, evaluating variant immune evasion, and validating new detection methods, PRNT and microneutralization assays still have irreplaceable roles [4,5,13,14].

Paper-based biosensors are more suitable for rapid initial screening of antibody levels after vaccination, primary-level testing, observation of population immune trends, and immune monitoring in resource-limited areas [104,105,106,107,108]. Their rapid, low-cost, portable, and easy-to-operate nature enables them to support broader population monitoring, especially suitable for use in communities, schools, border checkpoints, temporary vaccination sites, and remote areas [11,12,20].

For low- and middle-income countries, the significance of paper-based biosensors is even more prominent. These areas often face insufficient laboratory infrastructure, a shortage of professional personnel, difficulty in sample transportation, and limited testing funds, while the burden of infectious diseases is relatively heavy [11,63]. Paper-based platforms can help expand the accessibility of immune monitoring by lowering detection thresholds, narrowing the gap in vaccine immune evaluation capabilities between high-income regions and resource-poor regions, thereby promoting global vaccine equity [19,20,63].

**Figure 4:**
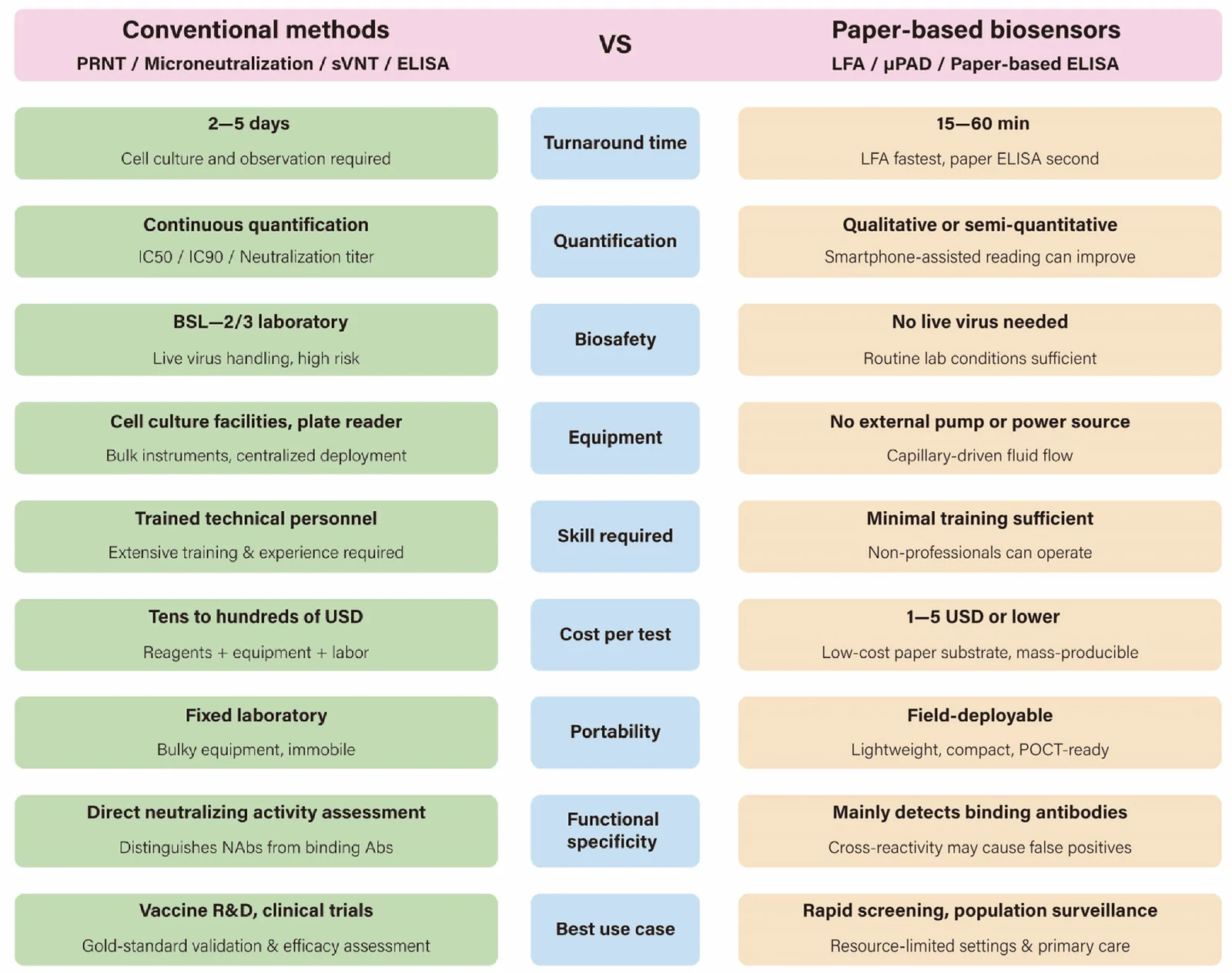
Comparison of traditional methods with paper-based sensors.

As shown in Figure 4, conventional neutralization assays and paper-based biosensors exhibit complementary strengths and limitations in terms of turnaround time, quantification capability, biosafety, equipment requirements, cost, portability, functional specificity, and application scenarios. Conventional neutralization tests are more suitable for confirmatory testing, scientific research, and standard method validation; paper-based biosensors are more suitable for field screening, primary monitoring, and large-scale population surveillance. The future development direction is not simply to replace conventional methods with paper-based detection, but rather, on the basis of rigorous validation and standardization, through antigen engineering, signal amplification, intelligent readout, AI-assisted analysis, and international standardization system construction, to improve the sensitivity, specificity, and quantitative capability of paper-based platforms, making them an important complement to traditional neutralization tests, and better serving vaccine-induced nAb monitoring, booster immunization strategy formulation, and global public health preparedness and response [25,42,43,107].

## 8. Challenges in Paper-Based Antibody and Surrogate Neutralization Assessment

### 8.1. Standardization and Quantitative Accuracy

Sensitivity, specificity, precision, reproducibility, linearity, stability, matrix interference, and lot-to-lot consistency are important parameters for evaluating the analytical performance of paper-based biosensors. These parameters are not specific to nAb detection, but they determine whether paper-based platforms can provide reliable and reproducible results. However, satisfactory analytical performance alone cannot demonstrate that the detected signal reflects functional neutralization. Therefore, paper-based blocking or neutralization-related assays also require biological validation to establish the relationship between the measured binding or inhibition signal and appropriate functional or clinical reference methods.

Insufficient standardization is one of the main problems limiting the deployment and regulatory approval of paper-based neutralizing antibody (nAb) detection. Detection results from paper-based platforms are easily affected by multiple factors including paper-based materials, reagent immobilization methods, signal output modes, result reading methods, and production batches. Different types of paper-based materials, such as nitrocellulose membranes, filter paper, and chromatography paper, differ in pore size, thickness, surface roughness, and hydrophilicity, and these differences further affect liquid flow rates, protein adsorption efficiency, and immune reaction processes [109]. Taking LFA as an example, material differences may change the residence time of immune complexes at the detection line; in μPADs, if processing techniques such as wax printing, laser cutting, or photolithography cannot ensure consistency in channel dimensions, the reproducibility of detection results will also be affected [109,110].

Reagent immobilization methods and their stability are also important factors affecting detection performance. Paper-based nAb detection requires immobilizing viral antigens, host receptor proteins, or capture antibodies on the paper surface, and different immobilization strategies—such as physical adsorption, covalent coupling, and affinity binding—each affect protein binding quantity, spatial conformation, and biological activity [111]. At the same time, immobilized proteins may become inactivated when temperature, humidity, or light conditions change, thereby reducing detection sensitivity or even producing false negative results. It is therefore necessary to establish standardized processes covering material screening, protein immobilization, drying preservation, transportation management, and control line design to ensure the stability and consistency of product performance.

Quantitative accuracy is also an important challenge facing current paper-based nAb detection. Conventional live-virus neutralization tests and pseudovirus neutralization tests can provide continuous titer data such as IC50, IC90, or NT50, while most paper-based platforms still primarily provide qualitative or semi-quantitative results [73,111]. The relationship between colorimetric signal and antibody concentration is often not linear, and the “hook effect” may occur in high-concentration samples, limiting the quantitative detection range [112]. In addition, visual interpretation is easily affected by lighting conditions and personal experience; even with smartphone or AI image analysis technology, unified shooting environments, hardware devices, and algorithm evaluation standards are needed [113]. Future paper-based detection platforms should further introduce standard reference sera, internal calibration zones, on-chip quality control modules, and unified result reporting units to better align detection results with traditional neutralization tests [76,77].

A critical gap in current standardization efforts is the lack of widely adopted international reference materials for nAb detection on paper-based platforms. The WHO International Standard for anti-SARS-CoV-2 immunoglobulin (NIBSC 20/136 [114]) was established to harmonize antibody titer reporting across assays, enabling results from different platforms to be expressed in standardized International Units (IU/mL) rather than arbitrary units [76,78]. However, the application of this standard to paper-based biosensors remains limited; most paper-based platforms report results as qualitative bands, signal-to-cut-off ratios, or arbitrary titers that cannot be directly traced to the IS. Furthermore, WHO International Standards are largely unavailable for viruses beyond SARS-CoV-2, such as influenza, dengue, Zika, and monkeypox, leaving paper-based nAb detection for these targets without a common calibration anchor. The development and adoption of virus-specific reference panels, including panels covering multiple variants and serotypes, would significantly improve inter-assay comparability and facilitate regulatory evaluation.

External quality assessment (EQA) programs represent another essential but underdeveloped component of standardization for paper-based nAb detection. EQA schemes, such as those coordinated by UK NEQAS or the College of American Pathologists (CAP) for serological testing, provide periodic proficiency testing that helps identify systematic biases and inter-laboratory variability. However, existing EQA programs are predominantly designed for laboratory-based serological assays and do not yet include paper-based POCT formats. Establishing EQA programs tailored to paper-based nAb detection—accounting for the unique challenges of point-of-care deployment such as variations in sample application, environmental conditions, and reader interpretation—would strengthen quality assurance and build confidence among regulators and end users.

Manufacturing standardization also presents significant challenges. Paper-based diagnostic devices must comply with quality management systems such as ISO 13485 (medical devices—quality management systems) [108], yet the inherent variability of cellulose-based substrates complicates consistent production. Nitrocellulose membranes from different suppliers—or even different lots from the same supplier—can exhibit variations in capillary flow rate, protein binding capacity, and background signal, all of which directly affect assay performance [109,111]. The REASSURED criteria (Real-time connectivity, Ease of specimen collection, Affordable, Sensitive, Specific, User-friendly, Rapid and robust, Equipment-free, Deliverable to end users) proposed by Land et al. provide a useful framework for evaluating POCT devices in resource-limited settings [115], but few paper-based nAb platforms have been systematically assessed against these criteria. Establishing minimum performance specifications aligned with REASSURED, along with standardized lot-release testing protocols and incoming material qualification procedures, would represent a meaningful step toward reproducible manufacturing and regulatory compliance.

### 8.2. Cross-Reactivity and Specificity

Cross-reactivity is another important factor affecting the specificity of paper-based nAb detection and is especially common between viruses with similar antigen structures. Cross-reactivity refers to the ability of antibodies induced by one virus or serotype to recognize and bind to the antigen of another related virus, leading to detection results that deviate from the true situation. Flavivirus family viruses are the most typical example of this problem; the envelope protein structures of different serotypes of DENV, ZIKV, JEV, and other flavivirus members have high similarity, and extensive serological cross-reactions are therefore prone to occur [87].

Since many paper-based detection platforms primarily rely on binding reactions between antigens and antibodies, cross-reactivity may produce false-positive signals, thereby overestimating the level of target virus antibodies. For example, in DENV-endemic areas, antibodies generated from previous ZIKV infection or JEV vaccination may bind to DENV antigens, but this binding does not mean the host has DENV-specific neutralizing capability. More noteworthy is that in the DENV infection process, some cross-reactive non-nAbs may also promote viral infection through antibody-dependent enhancement (ADE) [87]. If paper-based detection cannot effectively distinguish truly protective nAbs from cross-reactive non-nAbs, its results may mislead vaccine protective efficacy assessment and clinical risk judgment.

Similar situations also exist in orthopoxvirus and influenza virus detection. Monkeypox virus is antigenically similar to vaccinia virus and can easily cause confusion in serological detection results, and selecting species-specific antigens such as MPXV A29L helps improve the ability to distinguish between the two [60]. For influenza virus, cross-antibodies against the hemagglutinin stalk or conserved neuraminidase epitopes may indicate broad-spectrum protection but may also mask specific immune responses between different strains, so analysis and interpretation need to be combined with specific application scenarios.

The key to reducing cross-reactivity lies in optimizing antigen design and detection strategies. For example, using more specific antigen regions such as flavivirus envelope protein domain III (EDIII) can reduce interference from conserved region non-nAbs [89,90,116]. Multiplex detection modes can simultaneously detect multiple related viruses or different serotype antigens and assist in determining antibody origin based on signal distribution characteristics. Competitive or blocking detection strategies can directly assess the ability of antibodies to inhibit virus-receptor binding, thereby reducing the impact of non-neutralizing cross-antibodies. Even so, paper-based platforms currently still cannot completely avoid cross-reactivity, so result interpretation still needs to be combined with epidemiological background, vaccination history, previous infection history, and the co-circulation of related viruses for comprehensive judgment. The major cross-reactivity considerations among antigenically related viruses and the corresponding mitigation strategies are summarized in Table 5.

### 8.3. Clinical Validation and Regulatory Translation

Clinical validation is an indispensable step in the transition of paper-based antibody biosensors from laboratory research to practical application. The validation strategy should be determined according to the type of antibody response measured and the intended use of the assay. Binding-antibody assays, receptor-blocking assays, and surrogate neutralization assays reflect different biological signals and therefore require different performance claims and reference methods. Analytical validation should include sensitivity, specificity, precision, reproducibility, linearity, limit of detection, limit of quantification, hook effect, matrix interference, cross-reactivity, storage stability, lot-to-lot variation, user-to-user variation, and robustness under field conditions. For paper-based assays intended to reflect receptor blocking or surrogate neutralization, these analytical results should be further evaluated through correlation and agreement analyses with PRNT, VNT, pVNT, or a validated sVNT [25,31,68,71,76,77,78,96,97,112,115,117].

The first challenge in clinical validation is the selection of appropriate reference standards. PRNT and microneutralization assays are considered to be important reference methods for functional nAb detection, but they are operationally complex, have long detection cycles, and results may vary across laboratories. In comparison, sVNT and pseudovirus neutralization tests offer greater operational feasibility, but their consistency with the gold standard may be affected by factors such as virus strain type, antibody titer levels, and the characteristics of the tested population [73,77]. Different choices of reference methods may therefore directly affect the performance evaluation results of paper-based detection. In the future, it is necessary to establish clear, unified, and reproducible reference systems around different virus targets, while strengthening consistency research between different reference methods.

Beyond population diversity, the design of clinical validation studies for paper-based nAb detection requires careful attention to several methodological considerations. Sample size calculations should account for the expected prevalence of nAb positivity in the target population and the desired precision around sensitivity and specificity estimates; as a practical benchmark, the WHO Target Product Profile for SARS-CoV-2 antibody assays recommends ≥90% sensitivity and ≥95% specificity relative to PRNT. Blinded, head-to-head comparison with the reference standard is essential to avoid interpretation bias, and multi-center studies across geographically diverse sites help capture variability in circulating viral strains and population immune backgrounds. Concordance should be evaluated using multiple complementary metrics: Cohen’s kappa for categorical agreement, Spearman or Pearson correlation for quantitative titer comparison, and receiver operating characteristic (ROC) analysis to determine optimal cut-off values. Reporting only sensitivity and specificity without these more nuanced analyses may overstate or understate the true diagnostic performance of paper-based platforms. Furthermore, longitudinal clinical validation—assessing the ability of paper-based tests to track nAb dynamics over months post-vaccination—remains largely absent in the current literature but is critical for applications such as booster dose timing decisions.

The regulatory translation stage also requires paper-based detection products to have a clear application positioning and complete performance evidence. If used for community screening, the focus is more on operational convenience, detection speed, and sufficient sensitivity; if used for individualized booster immunization decisions, higher quantitative accuracy and clear clinical decision thresholds are needed [115]. Under regulatory frameworks such as EU IVDR or FDA, in vitro diagnostic products used for nAb monitoring need to submit data on precision, reproducibility, interfering factors, cross-reactivity, and clinical consistency [117]. Even after market launch, comprehensive batch quality management, laboratory proficiency testing, and post-market performance monitoring systems are still needed to continuously evaluate and optimize product performance to ensure long-term stability and clinical application value [115].

A more detailed examination of specific regulatory frameworks reveals the complexity of bringing paper-based nAb detection products to market. Under the U.S. FDA framework, paper-based nAb assays would typically follow either the 510(k) pathway—requiring demonstration of substantial equivalence to a legally marketed predicate device—or the De Novo classification pathway for novel device types without an existing predicate. Both pathways require analytical validation (including precision, accuracy, limit of detection, linearity, and interference studies) and clinical validation demonstrating acceptable concordance with an established reference method. Under the European In Vitro Diagnostic Regulation (EU IVDR 2017/746 [118]), which replaced the IVD Directive in May 2022, nAb detection devices are likely classified as Class C or Class D, given their role in assessing immune status for serious infectious diseases. This classification requires conformity assessment by a Notified Body, submission of clinical performance evidence from multiple clinical sites, and ongoing post-market surveillance including periodic safety update reports. In China, the National Medical Products Administration (NMPA) requires registration of in vitro diagnostic reagents, with nAb detection products classified as Class III (high-risk) medical devices necessitating rigorous clinical trial data from at least three clinical sites. These divergent regulatory requirements create a fragmented landscape for manufacturers seeking global market access, and harmonization of regulatory expectations across jurisdictions remains an important unmet need.

Paper-based biosensors have shown advantages of rapid, low-cost, and field-friendly operation in the area of nAb monitoring, but there is still some distance before they can widely enter clinical diagnosis and public health systems. Standardization, cross-reactivity control, quantitative detection capability improvement, and high-quality clinical validation are still the key directions for future development. This requires collaboration among academia, diagnostic companies, regulatory agencies, and public health departments to promote unified standard formulation, real-world validation, and quality control system improvement, so that paper-based nAb detection becomes an important complementary tool for traditional neutralization tests, better serving vaccine immune monitoring and global public health preparedness and response.

### 8.4. Real-World Implementation and Deployment Challenges

At present, the real-world implementation of paper-based antibody tests is uneven. Binding-antibody LFAs are relatively mature and widely used as rapid serological tools, whereas paper-based assays that can reliably approximate functional neutralization remain less established and require stronger clinical validation. Therefore, the goal of developing new paper-based nAb assays should not be limited to producing another rapid antibody test, but should be to provide clearly defined advantages in intended use, such as decentralized screening, longitudinal immune monitoring, variant-aware surveillance, multiplex detection, and correlation with functional neutralization standards.

After a paper-based nAb detection product has obtained regulatory clearance, its successful deployment in real-world settings faces substantial practical challenges that are often underestimated during the laboratory development phase. Supply chain reliability is a primary concern: paper-based diagnostic devices depend on a multi-component supply chain encompassing nitrocellulose membranes, conjugate pads, absorbent pads, labeled nanoparticles, and recombinant antigens, many of which are sourced from specialized suppliers with limited manufacturing capacity. Disruptions in the supply of any single component—as experienced globally during the COVID-19 pandemic—can halt entire production lines. Establishing dual-source strategies, maintaining strategic material reserves, and developing standardized component specifications that allow substitution without revalidation are critical for sustainable large-scale deployment.

End-user training and result interpretation represent another layer of implementation challenge. While paper-based assays are often described as “user-friendly” or “requiring minimal training,” studies have shown that operator variability in sample application volume, reading time, and visual interpretation can significantly affect result accuracy. For colorimetric LFAs, visual reading is inherently subjective and can be influenced by ambient lighting, color perception differences among users, and the reader’s level of experience. Even with smartphone-based readers, variations in camera models, shooting angles, and ambient conditions can introduce measurement inconsistency [33,119]. Standardized training protocols, competency assessment programs, and the inclusion of internal procedural controls on each test device are necessary to minimize operator-dependent variability in field settings.

Integration with health information systems is increasingly important for paper-based nAb detection deployed in public health programs. Test results need to be captured, transmitted, and aggregated in formats compatible with national health databases and immunization registries. This requires not only digital connectivity (e.g., smartphone apps with QR code-based result logging) but also interoperability with established health data standards such as HL7 FHIR (Fast Healthcare Interoperability Resources). Data governance frameworks must address patient consent, data ownership, cybersecurity, and cross-border data transfer regulations, particularly when results from multiple countries are aggregated for regional immune surveillance. The absence of standardized digital interfaces for paper-based POCT currently limits their integration into existing health information architectures.

Accordingly, the development of paper-based neutralization-related assays should not be limited to producing another rapid and low-cost antibody test. To provide meaningful advantages over existing serological methods, these platforms should offer one or more additional functions, such as compatibility with finger-prick blood, low sample consumption, reliable semi-quantitative or quantitative results, longitudinal monitoring, multiplex detection of variants or serotypes, integration with decentralized data systems, or standardized correlation with functional neutralization methods. Real-world studies should evaluate not only analytical performance but also invalid-test rates, user errors, environmental stability, workflow integration, accessibility, and the cost of obtaining a clinically useful result. Health economic evaluation is also important for the large-scale application of paper-based antibody testing. Although the unit cost of paper-based devices is generally low, the costs of readers, distribution, operator training, quality control, data management, confirmatory testing, and incorrect results should also be considered. Therefore, cost-effectiveness should be evaluated according to the cost of obtaining a reliable and clinically interpretable result rather than the cost of the paper-based device alone.

Particular attention must be given to implementation in low- and middle-income countries (LMICs), where the need for decentralized nAb monitoring is most acute but where deployment barriers are also most pronounced. High ambient temperatures and humidity in tropical regions can compromise the stability of immobilized proteins and labeled conjugates on paper-based devices, reducing shelf life and assay performance. Cold chain distribution, while less demanding than for mRNA vaccines, may still be required for certain reagent components. Additionally, the regulatory infrastructure in many LMICs may lack the capacity to conduct independent evaluation of paper-based nAb products, creating reliance on WHO Prequalification (PQ) or stringent regulatory authority approvals as proxies for quality assurance. Expanding the WHO PQ pathway to include paper-based nAb detection devices, and building regional regulatory capacity for in-country evaluation, are important steps toward equitable access to these technologies. Ultimately, successful real-world implementation of paper-based nAb detection requires a systems-level approach that addresses manufacturing scalability, supply chain resilience, workforce training, digital integration, health economics, and regulatory capacity in tandem, rather than treating these as isolated challenges.

## 9. Future Perspectives

Compared with existing reviews that mainly summarize the fabrication, signal amplification, or general diagnostic applications of paper-based biosensors, this review focuses more specifically on the use of paper-based platforms for monitoring virus-specific antibody responses, especially their potential and limitations in reflecting neutralizing activity. The key question is no longer only whether antibodies can be detected on paper, but whether the detected signal represents binding antibodies, receptor-blocking antibodies, or functional neutralizing antibodies (nAbs). Therefore, future development should not only improve device sensitivity and portability, but also address the biological interpretation, clinical validation, regulatory pathway, and real-world applicability of paper-based antibody monitoring.

### 9.1. Multiplex Detection of Multiple Viral Antibodies

One important future direction for paper-based neutralizing antibody (nAb) detection is the shift from single-virus, single-antibody testing toward simultaneous detection of multiple viruses, multiple variant strains, and multiple immune markers. Most existing paper-based detection platforms are designed for one particular virus or one antigen target. For example, SARS-CoV-2 detection typically centers on the RBD–ACE2 blocking reaction, whereas influenza, dengue, and Zika virus detection relies more on specific viral antigens or serotype antigens for antibody screening. In real-world public health settings, however, individuals may face simultaneous exposure to multiple viruses or receive vaccines against different viruses and variant strains. A single test result is therefore insufficient to fully capture an individual’s or a population’s immune status.

Multiplex detection platforms can measure multiple viral antibodies or antibody subtypes on a single paper-based device, providing a more complete immune profile. For SARS-CoV-2, future tests should not only assess nAbs against the ancestral strain but also detect those against variants such as Omicron, thereby gauging how broadly vaccine-induced immunity covers different variants. Song et al. developed a handheld NIR-to-NIR platform for on-site evaluation of protective nAbs against both the SARS-CoV-2 ancestral strain and the Omicron variant, showing that portable platforms hold promise for variant-specific immune assessment [29]. For influenza virus, multiplex detection can incorporate HA, NA, and different subtype antigens to assess the breadth of vaccine-induced antibodies. For flaviviruses such as DENV and ZIKV, multiplex detection is especially important because pronounced cross-reactivity exists among these viruses, and single-antigen testing is prone to misclassification. Nguyen et al. noted significant cross-reactivity in immune responses following DENV, JEV, and ZIKV infection, which affects vaccine evaluation and serological interpretation [67]. By arranging antigens from different viruses or serotypes on the same device, multiplex paper-based platforms can help distinguish virus-specific antibodies from cross-reactive ones.

From a technical standpoint, μPADs are better suited for multiplex detection than traditional LFAs. μPADs can achieve spatial separation of different sample paths, antigen regions, and signal outputs through multi-channel, multi-reaction-zone, and three-dimensional structural designs. Noviana et al. pointed out that μPADs offer considerable flexibility in structural design, enabling complex fluidic control through hydrophilic channels and hydrophobic barriers [20]. The three-dimensional μPADs constructed by Jeon et al. further showed that three-dimensional structures improve detection stability and spatial utilization on paper-based platforms [24]. In addition, although the autonomous lab-on-paper platform reported by Yin et al. was primarily designed for multiplex CRISPR-based SARS-CoV-2 diagnosis, its approach to automated fluidic control and multiplex detection can also inform the development of multiplex antibody assays [66].

Multiplex detection nonetheless introduces new challenges. First, cross-reactivity may exist among different antigens, particularly for antigenically similar viruses such as DENV, ZIKV, and JEV, and detection specificity must be improved through careful antigen design and competitive or blocking formats [67]. Second, signal crosstalk may occur among multiple detection zones, and colorimetric, fluorescent, or electrochemical signals from different test lines or spots must be clearly distinguishable. Third, multiplex detection generates larger volumes of data, making it difficult to interpret complex results by visual inspection alone. Future multiplex paper-based platforms therefore need to incorporate image recognition, machine learning, and standardized algorithms to enable automated signal analysis and integrated multi-parameter interpretation. Eryilmaz et al. developed a paper-based multiplex serological detection platform combined with machine learning for monitoring SARS-CoV-2 immune status, offering a useful framework for the future development of multi-virus antibody detection [120].

The development of liquid-biopsy biosensors also provides useful experience for the translation of paper-based antibody platforms. Both fields require reliable detection in complex biological samples, quantitative analysis of low-abundance targets, multiplex detection, standardized pre-analytical procedures, digital signal processing, and validation in heterogeneous clinical populations. Experience from liquid-biopsy research indicates that improvements in analytical sensitivity alone are insufficient for clinical translation. Paper-based antibody biosensors also require reliable calibration, appropriate reference materials, quality control procedures, standardized data collection, and evidence that the measured results can contribute to clinical or public health interpretation. Recent photonic and multimodal liquid-biopsy sensors further demonstrate the potential of multiplex detection and algorithm-assisted signal interpretation, while emphasizing the importance of assay traceability and clinical validation [121].

Overall, multiplex detection will transform paper-based nAb platforms from “single-point screening tools” into “comprehensive immune profiling tools.” Future research should focus on optimizing antigen combinations, spatial zoning designs, signal encoding strategies, and algorithmic interpretation models, enabling paper-based platforms to simultaneously assess different viruses, variant strains, antibody types, and immune states, thereby better serving vaccine evaluation and public health surveillance.

### 9.2. Integration with Digital Health and AI

Another important future direction for paper-based nAb detection is deep integration with smartphones, cloud databases, the Internet of Things (IoT), and artificial intelligence. The strength of traditional paper-based detection lies in its speed, low cost, and ease of use, but results often depend on visual inspection and are susceptible to ambient lighting, operator experience, and subjective interpretation. As smartphone cameras, portable readers, and AI-based image analysis advance, paper-based detection results can shift from subjective visual judgment to objective digital analysis.

Smartphones can serve as key reading terminals for paper-based detection platforms. Users photograph LFA test lines, paper-based ELISA colorimetric zones, or reaction areas of μPADs, and dedicated applications then analyze color intensity, fluorescence intensity, or image features. Compared with visual inspection, smartphone-based image analysis reduces subjective error and improves semi-quantitative and quantitative performance. Lee et al. reported a deep learning-assisted smartphone detection platform, showing that combining smartphones with AI enables sample-to-answer clinical assessment and improves automated interpretation of paper-based or portable detection results [122]. Another study by Lee et al. also showed that computer vision-assisted LFA improves diagnostic performance for influenza and SARS-CoV-2 detection, suggesting that AI image recognition can enhance result consistency on paper-based platforms [123].

The role of AI in paper-based nAb detection extends beyond image interpretation. Machine learning models can integrate serial detection results, vaccine type, time since vaccination, age, comorbidities, prior infection history, and epidemiological exposure data to predict antibody decay trends and the duration of immune protection. This capability is particularly important for monitoring vaccine-induced nAbs, because a single antibody measurement reflects immune status at only one time point, whereas a dynamic antibody curve more reliably indicates whether immune protection is declining, whether a booster dose is needed, and which populations should be prioritized for intervention. Eryilmaz et al. combined paper-based multiplex serological detection with machine learning to monitor SARS-CoV-2 immune status, illustrating that AI can further enhance the value of paper-based platforms in interpreting complex immunological data [120].

Beyond individual-level applications, digital health platforms can combine paper-based detection results with personal vaccination records, prior infection records, regional epidemic data, and variant surveillance data to form a more complete immune monitoring system. At the individual level, such a system can inform personalized booster recommendations; at the population level, it can identify areas or groups with insufficient immune protection. Research by Ardalan and Ignaszak on rapid SARS-CoV-2 biosurveillance tools also suggests that rapid detection technologies must be coupled with data systems to better serve public health surveillance and outbreak response [124].

Digitalization and AI integration, however, introduce new issues. First, image acquisition for paper-based detection must be standardized, including camera distance, lighting conditions, background color, and image calibration procedures. Second, AI models require sufficiently large and diverse training data to accommodate different sample types, populations, and detection batches. Third, individual antibody test results constitute sensitive health data, and uploading them to cloud-based systems demands attention to data security, privacy protection, and ethical governance. Future platforms should establish uniform data acquisition standards, algorithm validation frameworks, and privacy protection mechanisms to prevent clinical and public health applications from being compromised by algorithmic bias or data breaches.

In summary, AI and digital health technologies will shift paper-based nAb detection from a paradigm of “single, offline, manual reading” toward “continuous, networked, intelligent analysis.” This transition will not only improve detection accuracy and result interpretability but also facilitate individualized vaccine strategies and real-time public health decision-making.

### 9.3. Large-Scale Immunity Surveillance

A major ultimate application goal of paper-based nAb detection is to serve large-scale immune surveillance. Conventional nAb assays are accurate but depend on laboratories, cell culture, trained personnel, and long turnaround times, making them unsuitable for high-frequency, population-wide screening. Paper-based platforms, by contrast, offer low cost, speed, portability, and ease of use, making them better suited for immune surveillance in communities, schools, nursing homes, primary healthcare facilities, vaccination sites, and resource-limited settings.

The core value of large-scale immune surveillance lies in timely understanding of protective immunity levels across different populations. nAb levels after vaccination decline over time and vary substantially among individuals. Age, comorbidities, immune status, prior infection history, vaccine type, and dosing intervals can all influence antibody production and decay rates. Relying solely on standardized vaccination schedules is therefore insufficient to determine the true protective status of all individuals. Paper-based platforms can support more frequent and broader population-level antibody monitoring through their low cost and high accessibility.

In the SARS-CoV-2 field, paper-based detection has already shown practical value for post-vaccination immune surveillance. Tuells et al. used rapid LFIC and sVNT-ELISA to detect post-vaccination nAbs among healthcare workers, demonstrating that rapid detection methods are applicable to immune assessment in real-world populations [71]. In addition, the paper-based microfluidic surrogate virus neutralization assay developed by Mahmud et al. can detect SARS-CoV-2 nAbs without live virus, reflecting the trend of paper-based platforms toward on-site, semi-quantitative, and functional assessment [1]. If such assays are further optimized and standardized, they could be deployed for large-scale post-vaccination antibody surveillance.

Large-scale surveillance can also help public health authorities identify immunity gaps. For example, periodic nAb testing among older adults, immunocompromised individuals, healthcare workers, and high-exposure occupational groups can indicate whether booster immunization should be prioritized. During periods of variant predominance, multi-strain or multi-variant paper-based assays can evaluate how well existing vaccines protect against new variants. The platform developed by Song et al. for on-site evaluation of nAbs against both the ancestral strain and the Omicron variant suggests that future portable antibody detection can be integrated with variant surveillance to inform vaccine resource allocation and booster strategies [29].

Large-scale immune surveillance is equally important for viruses such as dengue, Zika, and influenza. Frequent antigenic drift of influenza viruses makes population-level antibody monitoring valuable for assessing vaccine strain matching and immune coverage. In regions where DENV and ZIKV co-circulate, distinguishing antibody responses induced by different flavivirus infections or vaccines is necessary to avoid misclassification caused by cross-reactivity [67]. Future paper-based platforms capable of simultaneous detection of multiple viruses, serotypes, and variants will be better suited to complex epidemiological scenarios.

To achieve large-scale immune surveillance in practice, however, paper-based platforms must still address standardization and quality control issues. Results from different manufacturers, batches, and reading methods must be comparable. Many current paper-based assays produce only qualitative or semi-quantitative results that cannot be directly mapped to IC50, IC90, or international standard units from conventional neutralization assays. Girl et al.’s evaluation of rapid LFAs and surrogate ELISAs indicated that rapid detection methods must be systematically compared with standard methods to establish their clinical and public health utility [68]. Before large-scale deployment, uniform calibrators, quality control systems, result grading criteria, and data reporting standards should therefore be established.

In sum, paper-based nAb detection can provide a low-cost, rapid, and decentralized technical foundation for large-scale immune surveillance. Its future value lies not only in “detecting antibodies” but also in using serial monitoring to identify immune decay, guide booster vaccination, assess breadth of vaccine protection, and supply real-time data for public health decision-making during emerging virus outbreaks.

### 9.4. Clinical Translation: Standardization, Validation, Regulation, and Real-World Implementation

Clinical translation is the main bottleneck for paper-based antibody and nAb detection. Many platforms show promising proof-of-concept performance, but clinical use requires more than high sensitivity under laboratory conditions. Four issues are particularly important: standardization, clinical validation, regulatory positioning, and real-world implementation.

First, standardization is essential. Paper-based assays are affected by paper pore size, thickness, hydrophilicity, protein adsorption, reagent immobilization, drying conditions, storage temperature, humidity, sample matrix, incubation time, and readout timing. These variables can alter flow rate, background signal, and binding efficiency. Ardakani and Hemmateenejad noted that μPADs still face challenges in sensitivity, stability, manufacturing consistency, real-sample applicability, and clinical translation [25]. Therefore, future studies should standardize materials, antigen or receptor immobilization, blocking conditions, storage procedures, sample volume, reading time, and result interpretation. For quantitative assays, calibration curves, reference materials, internal controls, and batch-to-batch quality control are indispensable.

Second, clinical validation should clarify what the assay actually measures. A paper-based assay that detects antigen-binding antibodies should not be directly interpreted as an nAb assay unless it has been validated against functional or surrogate neutralization methods. Validation should include analytical sensitivity, specificity, precision, linearity, limit of detection, matrix interference, cross-reactivity, and reproducibility. More importantly, it should involve diverse clinical samples from individuals with different infection histories, vaccination backgrounds, ages, immune statuses, and disease conditions. Comparisons with PRNT, pVNT, sVNT, or clinical protection data are needed to determine whether paper-based signals reflect functional neutralizing activity. Studies comparing rapid LFAs and surrogate ELISAs have already shown that different rapid methods may vary in accuracy and concordance [68,71].

Third, regulatory positioning should be defined early. Paper-based antibody tests may be used for research, epidemiological surveillance, post-vaccination immune monitoring, clinical decision support, donor screening, outbreak response, or home self-testing. Each intended use requires different evidence. A population screening tool may emphasize accessibility and cost, whereas a clinical decision-support test requires stronger accuracy and clinical evidence. Home self-testing also requires usability evaluation, clear instructions, and safeguards against misinterpretation. If digital or AI-assisted interpretation is used, software validation, algorithm version control, data privacy, and cybersecurity must also be considered. Zhu et al.’s TimePAD illustrates how deep learning can improve μPAD signal analysis [72], but such tools would require formal validation before clinical deployment.

Finally, real-world implementation must be tested under actual operating conditions. Field use may involve non-specialist users, finger-prick whole blood, variable temperature and humidity, limited equipment, unstable supply chains, and inconsistent internet access. Therefore, implementation studies should evaluate not only sensitivity and specificity, but also workflow feasibility, training needs, turnaround time, user error, transport stability, cost per test, waste disposal, and integration with public health reporting systems. Although nanomaterials, fluorescent labels, SERS, and AI-based readers can improve analytical performance, they may also increase cost and operational complexity [31]. Future platforms should therefore balance sensitivity, affordability, robustness, and usability. Only when paper-based antibody assays are manufacturable, standardized, clinically validated, interpretable, affordable, and regulatable can they move from laboratory prototypes to reliable tools for immune monitoring and public health decision-making.

## 10. Conclusions

Paper-based biosensors provide accessible and adaptable platforms for monitoring antiviral antibody responses. Depending on the assay format, lateral flow assays, microfluidic paper-based analytical devices, and paper-based enzyme-linked immunosorbent assays may measure antigen-binding antibodies, receptor-blocking activity, or surrogate neutralization. Antigen-binding signals primarily indicate the presence and magnitude of virus-specific humoral responses and should not be interpreted as direct evidence of neutralizing activity. Competitive or receptor-blocking formats are more closely related to antibody function, but they remain simplified surrogates and require validation against appropriate functional reference methods [1,10,12,25,68].

The principal value of paper-based antibody assays lies in rapid screening, longitudinal monitoring, decentralized testing, and population-level immune surveillance, particularly in field settings and resource-limited areas [11,12,17,20]. However, most current paper-based assays remain qualitative or semi-quantitative, may be affected by cross-reactivity, matrix interference, material variability, and reader-dependent interpretation, and cannot directly provide functional neutralization parameters such as IC50, IC90, or NT50 [25,31,67,68]. Accordingly, paper-based biosensors should be regarded as complementary tools rather than replacements for the plaque reduction neutralization test, virus neutralization test, or pseudovirus neutralization test, which remain necessary for confirmatory functional assessment, determination of protective thresholds, and rigorous vaccine evaluation [4,5,13,14]. Future translation will depend on clear assay classification, standardized calibration, virus- and variant-specific antigen design, cross-reactivity control, prospective clinical validation, and intended-use-specific regulatory evidence. Multiplex detection and digital readout may further improve accessibility and result interpretation, but these technologies must also undergo independent analytical and clinical validation before supporting individual clinical decisions [21,22,72].

## Figures and Tables

**Table 1:** Comparison of conventional and surrogate neutralization-related assays.

Assay	What It Measures	Functional Relevance to Neutralization	Main Limitation
PRNT/VNT	Reduction in infectious viral plaques or cytopathic effect by serially diluted serum	Gold standard; directly reflects functional neutralizing capacity	Live virus, BSL-2/3 containment, 2–5 days, operator-dependent, low throughput
pVNT	Inhibition of pseudoviral (e.g., luciferase- or VSV-based) entry into reporter cells	High; approximates functional neutralization without authentic pathogen	Still requires cell culture; pseudotype construction; not identical to wild-type virus
sVNT/receptor-blocking ELISA (e.g., cPass)	Blocking of virus-receptor engagement (e.g., RBD-ACE2) by serum antibodies	Surrogate for neutralizing activity; correlates with PRNT	Relies on a single blocked epitope/step; may miss other neutralization mechanisms; needs per-variant validation
Binding ELISA/standard serology	Total antigen-binding antibodies (IgG/IgM)	Low/indirect; only a correlate of immunity	Does not assess function; levels may not predict protection or cross-neutralization

**Table 2:** General performance of paper substrates used in paper-based antibody biosensors.

Substrate	Pore Size	Flow Rate	Protein Immobilization	Background Signal	Mechanical Strength	Batch Consistency	Suitable Platform
Nitrocellulose membrane	0.2–15 μm (commonly 0.45–8 μm for LFA)	Low-moderate; capillary-controlled	High (hydrophobic/electrostatic capture)	Low-moderate (reduced by blocking)	Low (brittle, weak when wet)	Moderate (lot-to-lot variability)	LFA, dipstick
Filter paper (e.g., Whatman)	~2.5–30 μm (Grade 1 approx 11 μm)	High	Moderate (hydroxyl groups; needs activation)	Moderate	Moderate (softens when wet)	Good (well standardized)	muPAD, paper-based ELISA, 3D
Chromatography paper	~5–13 μm	Moderate-high	Moderate	Low-moderate	Moderate	Good	muPAD
Cellulose paper	Variable, fibrous	Moderate	Moderate (abundant OH groups)	Moderate	Moderate-high	Moderate	muPAD, origami, ELISA
Functionalized/treated paper	Depends on base + treatment	Tunable	High (via chemical modification)	Low-moderate	Tunable	Depends on process	LFA, muPAD, ELISA

**Table 3:** Virus-specific neutralization-related antigens and implications for paper-based assay design.

Virus	Neutralization-Related Antigen/Component	Relevance to Assay Design	Key Interpretation Issue
SARS-CoV-2	Spike RBD/S2/ACE2-binding site	RBD-ACE2 blocking is the dominant neutralization mechanism, ideal for sVNT	High RBD-binding may not equal broad nAb; variant escape (e.g., Omicron) lowers correlation
Influenza	Hemagglutinin (HA) head + stalk; neuraminidase (NA)	HA mediates entry; HA-stalk and NA are broadly conserved	Antigenic drift/shift requires updated antigens; stalk antibodies broader but lower titer
Dengue (DENV)	Envelope (E) protein, domain III (EDIII)	Serotype-specific neutralizing epitopes concentrate in EDIII	Four serotypes; cross-reactive non-neutralizing antibodies imply ADE risk; serostatus matters
Zika (ZIKV)	Envelope (E), EDIII	Shares flavivirus cross-reactive epitopes with DENV/JEV	Difficult to distinguish ZIKV from DENV nAbs; cross-reactivity confounds specificity
Mpox (MPXV)	A29L, A35R, B6R, M1R (entry/fusion)	A29L-based sVNT is feasible	Antigenic similarity to vaccinia/other orthopox requires species-specific antigen selection

**Table 4:** Performance-oriented comparison of paper-based platforms for vaccine-induced nAb monitoring.

Platform	Representative Assay Mode	Sensitivity and Detection Limit	Specificity	Quantitative Capability	Correlation with Gold-Standard Assays	Key Translational Limitation
LFA	Binding-antibody LFA or competitive/blocking LFA, depending on antigen and probe design.	Moderate overall; improved by fluorescence, quantum dots, SERS, nanozymes, or silver enhancement. Detection limit is highly label- and reader-dependent.	Moderate to high when antigen selection is optimized; vulnerable to cross-reactivity in antigenically related viruses.	Mainly qualitative or semi-quantitative; portable readers and smartphone/AI analysis can improve signal quantification.	Variable. Stronger for competitive or receptor-blocking formats than for binding-antibody formats; requires validation against PRNT, pVNT, or sVNT.	Often reflects binding or surrogate blocking rather than true functional neutralization; limited dynamic range and hook-effect risk.
μPADs	Paper microfluidic immunoassay with multi-channel, competitive, blocking, or surrogate neutralization design.	Moderate to high; can be enhanced by nanomaterials, fluorescence, chemiluminescence, or electrochemical readouts. Detection limit is device- and flow-control dependent.	Moderate to high; multiplex layouts can improve discrimination, but antigen cross-reactivity and channel crosstalk remain concerns.	Semi-quantitative to quantitative when coupled with calibrated optical, electrochemical, or digital readouts.	Promising but still limited. Best supported when the device directly measures virus-receptor blocking; broader clinical correlation data are needed.	Fabrication reproducibility, evaporation, sample matrix effects, flow variability, and lack of standardized calibration restrict clinical translation.
Paper-based ELISA	Miniaturized paper ELISA for binding antibodies or competitive/blocking paper ELISA for surrogate nAb assessment.	Moderate to high due to enzymatic amplification; generally more sensitive than simple colorimetric LFA, but still assay-dependent.	High when antigen immobilization and washing are well controlled; binding formats may still capture non-neutralizing antibodies.	Semi-quantitative to quantitative with image analysis, portable readers, or time-resolved/AI-assisted readout systems.	Moderate to high for sVNT-like blocking formats; indirect for conventional binding ELISA formats.	More steps than LFA, including incubation and washing; protein immobilization, background control, and automation remain major barriers.

**Table 5:** Cross-reactivity considerations among antigenically related viruses.

Virus Group	Antigenic Relationship	Cross-Reactive Viruses	Risk	Mitigation Strategy
Flavivirus	High envelope-protein homology	DENV, ZIKV, JEV, WNV	False-positive binding/neutralization; ADE upon heterotypic infection	EDIII-specific antigen; competitive/blocking format; multiplex serotyping; PRNT confirmation
Orthopoxvirus	Conserved pox proteins (e.g., A29L, A35R)	MPXV, Vaccinia, Cowpox	False-positive due to conserved orthopox antigens	Species-specific antigens (A29L, M1R); differential peptide arrays
Influenza (subtypes)	Conserved HA stalk/NA	H1/H3; H5/H7, etc.	Underestimation of subtype-specific response	Head-domain-focused antigen; stalk-targeted design
SARS-CoV-2 vs. seasonal coronaviruses	Partial spike homology	OC43, HKU1, NL63, 229E	Low but possible cross-binding	RBD-specific antigen; blocking format

## Data Availability

No new data were created or analyzed in this study. Data sharing is not applicable to this article.
